# The Association of Periodontitis With Cardiovascular Disease Parameters: A Synthesis of Systematic Reviews

**DOI:** 10.1111/idh.12885

**Published:** 2026-02-24

**Authors:** Max G. P. Schoenmakers, Lotte P. M. Weijdijk, Eveline J. S. Willems, Fridus (G. A.) van der Weijden, Dagmar Else Slot

**Affiliations:** ^1^ Department of Periodontology Academic Center for Dentistry Amsterdam (ACTA) University of Amsterdam Amsterdam The Netherlands; ^2^ Department of Oral and Maxillofacial Surgery Amsterdam UMC and Academic Centre for Dentistry Amsterdam (ACTA), University of Amsterdam and the Faculty of Dentistry of the Vrije Universiteit Amsterdam Amsterdam The Netherlands

**Keywords:** cardiovascular diseases, cardiovascular events, causality, meta review, periodontitis, umbrella review

## Abstract

**Focused Question:**

What is the association of periodontal disease (PerioD) and cardiovascular diseases (CVD/CVE) as reported in existing systematic reviews (SRs)?

**Methods:**

MEDLINE‐PubMed and Cochrane‐CENTRAL databases were searched. Papers that primarily evaluate cardiovascular parameters of CVD and CVE in PerioD patients compared to non‐PerioD individuals were included. Data and conclusions as presented in the selected papers were extracted and the potential risk of bias was estimated. A descriptive analysis of the meta‐analyses of the selected studies was conducted. A citation analysis was performed, the Bradford Hill criteria were assessed and the acquired evidence was graded.

**Results:**

Independent screening of 446 reviews resulted in 19 eligible SRs. These were categorised into 13 reviews evaluating CVD and eight evaluating CVE. In total 27 meta‐analyses were obtained, the majority (78%) of reported risk ratios and odds ratios are estimated to show a negligible magnitude of the association of PerioD and CVD. For CVE, 46% of the values of the association are considered to be of small magnitude as emerging from 23 meta‐analyses. For factors such as gender, age, PerioD severity, smoking status and geographic region, the statistical significance and magnitude of the association varied. Given the results, a definitive confirmation of causality according to the Bradford Hill criteria was not attainable. With moderate certainty, a predominantly negligible to small magnitude of the association of PerioD and CVD/CVE was identified.

**Conclusion:**

Based on data collected from existing SRs, the association between PerioD and CVD/CVE was generally observed to be of negligible to small magnitude. Additionally, the data does not confirm potential causality.

## 1 Introduction

Periodontal disease (PerioD) is a chronic inflammatory disease affecting the tissues that support the teeth, which include the gums, bone and periodontal ligament [1, 2, 3]. Estimates suggest that between 20% and 50% of all individuals worldwide are affected by PerioD, with severe forms of the disease affecting approximately 11% of the global population [4, 5, 6]. PerioD is a major contributor to tooth loss (TL), accounting for 30%–35% of all cases. In addition to affecting dental health, PerioD can lead to difficulties with chewing, negative aesthetic changes and systemic inflammation, significantly impacting the quality of life for those affected [1, 2, 7]. Recent research has suggested that PerioD may have systemic effects, particularly on cardiovascular health, due to its association with various risk factors such as chronic inflammation and bacterial infections [4, 8, 9, 10].

Cardiovascular diseases (CVD/CVE) as defined by the World Health Organisation (WHO) [11], are a group of disorders affecting the heart and blood vessels and could be divided into CVD, including cerebrovascular disease, coronary heart disease (CHD), atherosclerotic cardiovascular disease (ACVD), peripheral vascular disease (PVD) and cardiovascular events (CVE) [11]. CVD, which is often asymptomatic, has the potential to result in acute CVE such as myocardial infarction (MI) and stroke, making it a prominent global cause of death [12, 13]. As human life expectancy continues to increase, the prevalence of chronic diseases, particularly CVD, also rises [12]. In 2019, CVD/CVE were globally the number one cause of death. Ischemic heart disease (IHD) was found to be the world's biggest killer, followed by stroke. Those two leading causes of death are accountable for 16% and 11% of all deaths respectively. Moreover, the fatal endings due to IHD is increasing since 2000 [13].

PerioD and CVD/CVE have some shared risk factors such as smoking, diabetes mellitus (DM), obesity, poor oral health and stress [4, 14]. Given the high prevalence of PerioD and CVD/CVE, as well as their shared risk factors, there is a growing interest in understanding the association between these two conditions. Numerous studies have shown the significant independent association between PerioD and CVD/CVE [9, 10, 15]. There is even the suggestion of a *two‐way* (*bidirectional*) relationship between PerioD and CVD/CVE [16]. In more detail, there is increasing evidence suggesting that PerioD may be a risk factor for CVD/CVE, such as coronary artery disease, stroke and MI [15]. However, a causal relationship has yet to be proven. Recently, Lavigne and Forrest [17] have published an umbrella review examining the possible causal relationship of PerioD to CVD by investigating systematic reviews (SRs) determining if periodontal therapy lowers the risk of CVE. The result of their review confirms the existence of an association. However, there was insufficient evidence to satisfy the Bradford Hill criteria [18] and to state that there is also a causal relationship between CVD/CVE and PerioD [17].

Despite the growing body of literature on the relationship between PerioD and CVD/CVE, the evidence remains elusive and the practical implication is unclear. Therefore, there appears to be a need for a comprehensive and critical appraisal of the available evidence concerning the association between PerioD and CVD/CVE and in addition estimate the potential causal link between these two conditions. Accordingly, the aim of this study was to prepare a synthesis of SRs (meta‐review) on the association of PerioD and CVD/CVE.

## 2 Methods

A protocol was developed a priori following the initial discussion between the members of the research team. The preparation and presentation of this meta‐review is in accordance with the Joanna Briggs Institute (JBI) methodological guideline [19], the Meta‐analysis of Observational Studies (MOOSE) guideline [20], the PRISMA guideline [21, 22] and the AMSTAR tool [23] to ensure the methodological quality of the review process and improve the strength of reporting. This study is registered at the International Prospective Register of Systematic Reviews (PROSPERO) by number CRD42023444999. The Institutional Review Board of the Academic Centre for Dentistry Amsterdam (ACTA) also provided approval with the following number: 2022‐74229.

### 2.1 Focused Question

A review question was formulated utilising the population, exposure, comparison, outcomes and study (PECOS) framework as follows [24].

What associations can be identified between CVD/CVE in individuals with PerioD as opposed to those without PerioD, based on information gathered from existing SRs?

- Patients: humans ≥ 16 years old
- Exposure: patients with PerioD
- Comparison: individuals without PerioD
- Outcome: parameters of CVD/CVE
- Study design: SRs

### 2.2 Search Strategy

As part of the search strategy, electronic databases, including MEDLINE‐PubMed, and special collections of Cochrane‐CENTRAL were systematically queried up to 21 September 2023. The structured search aimed to identify relevant SRs and meta‐analyses that address the association of PerioD on CVD. The comprehensive search was designed by two reviewers (MGPS and DES) to include all SRs that answered the focused question. Table 1 provides more details regarding the search approach employed. Additionally, references cited in the included studies were screened for supplementary SRs and the PROSPERO database was checked for ongoing reviews. No further unpublished work or grey literature was sought.

**TABLE 1:** Details regarding search terms. Search strategy used for MEDLINE‐PubMed.

**{[<exposure>] AND [<outcome>]}**
**<Exposure:>** <((“Periodontitis”[Mesh]) OR Periodontitis OR (periodontal disease) OR (periodontal diseas*) OR (periodontal infection) OR periodont*)>
**<Outcome:>** <((“cardiovascular diseases” [MesH]) OR (cardiovascular diseases) OR cardi OR (cardiac disease) OR stroke OR cerebro OR (cerebrovascular accident) OR stroke OR Atherosclerosis OR arthero* OR myocardial* OR (Myocardial ischemia) OR (myocardial disease) OR (chronic heart disease) OR (cardiovascular disease) OR cardio* OR (acute myocardial infarction) OR (coronary vascular disease) OR (peripheral arterial disease))> AND <(systematic review*) OR meta‐analysis OR (meta analysis) OR (umbrella review*)>

*Note:* The asterisk (*) was used as a truncation symbol. The search strategy was customised according to the database being searched.

### 2.3 Screening and Selection

Duplicate papers were identified and removed before the assessment of the SRs. Titles and, when available, abstracts of all SRs were screened. This was performed by three independent reviewers (MGPS, EJSW and LPMW) using the Rayyan web application [25, 26]. Titles and abstracts of all studies were read in detail and categorised as included, excluded or undecided using the inclusion criteria. The reviewers were blinded from each other's results during the two‐stage selection process. After the screening process, the search was unblinded and disagreements concerning eligibility were identified by Rayyan [25, 26]. Only when full agreement was reached between the three reviewers (MGPS, EJSW and LPMW) the paper was included. Discrepancies were discussed and resolved to reach consensus and in case of disagreement, a following discussion with a fourth reviewer (DES) made the final decision. Full‐text papers were obtained, further assessed and ultimately processed for data extraction when all the inclusion criteria were fulfilled. Updates of SRs were checked and the latest version was selected.

### 2.4 Inclusion and Exclusion Criteria

Studies that evaluated the association between PerioD and CVD/CVE relative to individuals without PerioD as outcome variable were included. A full‐text review of all the pertinent articles was completed, utilising the following detailed eligibility criteria:

- SRs with or without a meta‐analysis.
- Full‐text publications available in English or Dutch.
- SRs of studies conducted in humans that had at least two groups of individuals:
  ○ Evaluating a group of patients with PerioD of ≥ 16 years old.
  ○ Evaluating a group of individuals without PerioD of ≥ 16 years old.
  ○ No restriction was applied for the definition and severity of PerioD.
- Studies assessing CVD‐related outcomes as confirmed by any of the following:
  ○ CVD as defined by cerebrovascular disease, CHD, ACVD and PVD.
  ○ As the terms peripheral artery disease (PAD) and PVD are often used interchangeably, for this review, the parameter PAD was described along with PVD [27].
  ○ CVE as defined by stroke and MI.
- Data from a SR were taken into consideration if more than one original study contributed to the underlying evidence.

The exclusion criteria were as follows:

- Pregnancy.
- Apical periodontitis.
- Peri‐implantitis.
- DM or any other synonymous condition (such as metabolic diseases).
- CVD outcomes defined by surrogate markers, biomarkers or antibody levels.
- CVD outcomes derivatives such as hypertension (HT), atrial fibrillation (AF), arterial stiffness and carotid artery calcification.
- PerioD defined only by number of teeth or TL.
- Studies that primarily focused on the effect of periodontal treatment.

### 2.5 Data Analysis

#### 2.5.1 Assessment of Heterogeneity

The heterogeneity across studies was detailed according to the following factors:

- Methodological: variability in review approach, risk of bias assessment and analysis performed (descriptive and/or meta‐analysis).
- Clinical: subject characteristics, PerioD details, CVD/CVE details.

#### 2.5.2 Citation Analysis

To ascertain potential overlap among the primary clinical studies within the included SRs, a citation matrix was constructed. This matrix aimed to compile a comprehensive list of unique studies [28].

#### 2.5.3 Data Extraction

The papers that passed the screening and selection process and fulfilled the inclusion criteria were processed for data extraction. If studies examined a bi‐directional relationship between PerioD and CVD, only those data were extracted that evaluated PerioD as exposure variable and CVD/CVE as outcome. The included SRs were categorised into groups based on CVD/CVE outcomes as defined by the WHO [11], being parameters of CVD and CVE. This together with data extraction was performed by two independent reviewers (MGPS and LPMW) using a standardised data extraction form. Disagreements between the reviewers were solved by discussion. If disagreement persisted, a third reviewer (DES), was consulted. The following characteristics of the included studies were extracted: publication details, focused question, search results, number of included studies, details on CVD/CVE outcome and the conclusions from the original authors. Furthermore, the method of analysis, whether descriptive and/or involving meta‐analyses, was also documented. From the available meta‐analyses, the risk ratio (RR) or odds ratio (OR) and the corresponding 95% confidence interval and *p* value were extracted for both random‐ and fixed‐effects models. Additionally, statistical heterogeneity of the included meta‐analyses was extracted from tests of heterogeneity such as *I* ^2^, *Q*, *X* ^2^ and Ri together with the respective *p* values. When within an included SR‐performed subgroup analysis was performed for gender, age, smoking status, PerioD severity and/or study region, the corresponding data were also extracted. When feasible subgroup analysis on these patient and study characteristics was performed.

#### 2.5.4 Data Interpretation

As a guide for interpreting the magnitude of the association of the extracted RRs, values of 1.22, 1.86 and 3.00 were deemed indicative of small, medium and large magnitudes, respectively [29]. Additionally, the values of the ORs of 1.68, 3.47 and 6.71 were indicative of small, medium and large magnitudes, respectively [30]. Meta‐analyses outcomes resulting in a *p* < 0.05 were considered to be statistically significant.

Tests of heterogeneity resulting in a *p* < 0.1 were considered to be statistically significant. As a guide to evaluate the potential magnitude of inconsistency among primary studies, an I^2^ statistic of 0%–40% may indicate negligible levels of heterogeneity, 30%–60% may suggest moderate heterogeneity and 50%–90% may indicate substantial heterogeneity. Furthermore, an I^2^ statistic exceeding 75% was interpreted to indicate considerable heterogeneity [31].

### 2.6 Quality Assessment

The risk of bias was estimated independently by two reviewers (MGPS and LPMW) rating the reporting and methodological quality of the included SRs and meta‐analyses using a combination of items described by the PRISMA [21, 22] and the AMSTAR [23] checklist.

A list of 27 items was evaluated, each aspect of the reporting and methodological quality item score list was given a rating of a plus (+) for informative description of the item at issue and a study design meeting the quality standard, was assigned. Plus‐minus (±) was assigned if the item was incompletely described and minus (−) was used if the item was not described or unknown [32].

For the quality assessment score, individual items with a positive rating were summed to obtain an overall percentage score and a score of 100% was reached if all individual items received good ratings when these ratings were added together [32, 33]. As a guide to interpret the estimated risk of bias, a range of 0%–40% may indicate a high risk of bias, a range of 40%–60% may indicate a substantial risk of bias, a range of 60%–80% may indicate a moderate risk of bias and a range of 80%–100% may indicate a low risk of bias. Only SRs that included a meta‐analyses could achieve a full score of 100% [34].

### 2.7 Assessment of Causality

To evaluate potential causality, the Bradford Hill criteria [18] were employed, which offers a framework to evaluate the strength of evidence supporting a causal relationship between PerioD and CVD/CVE. The following nine criteria were assessed by two reviewers independently (MGPS and LPMW): strength of association, consistency, specificity, temporality, biological gradient, plausibility, coherence, experiment and analogy. Disagreements were resolved through additional discussion to reach a consensus. If a disagreement persisted, the judgement of a third reviewer (DES) was decisive.

### 2.8 Grading the ‘Body of Evidence’

In this synthesis of SRs, the evidence was evaluated using the Grading of Recommendations Assessment, Development, and Evaluation (GRADE) system [35] developed by the GRADE working group [36, 37]. The quality of the evidence was rated by two reviewers (MGPS and LPMW) as well as the strength of the recommendations according to the following aspects: study design, risk of bias, consistency and precision among outcomes, directness of results, detection of publication bias and magnitude of the association. Any disagreement between the two reviewers was resolved after additional discussion. If a disagreement persisted, then the judgement of a third reviewer (DES) was decisive.

## 3 Results

### 3.1 Search and Selection Results

**FIGURE 1:**
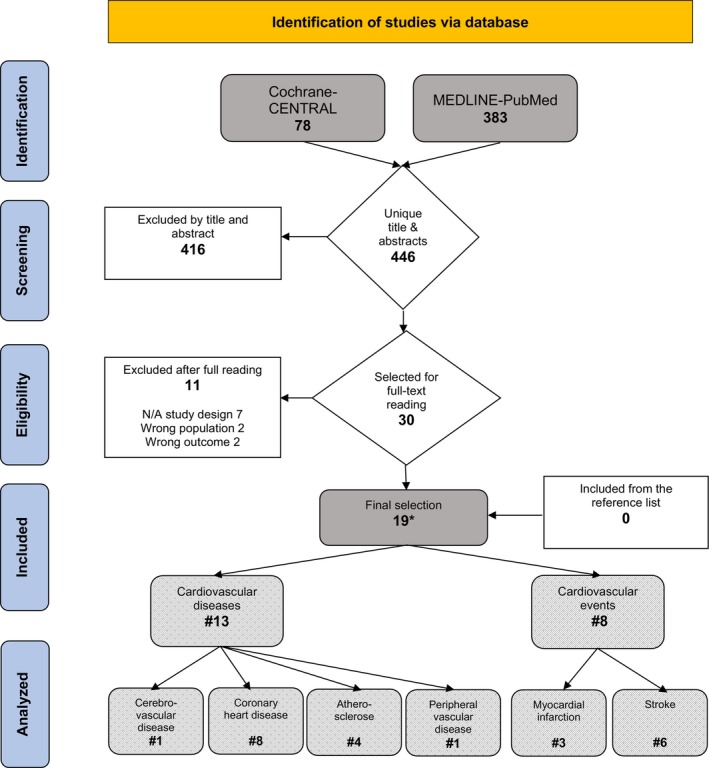
Search and selection results.

The search and selection process is illustrated in Figure 1. A total of 461 titles and abstracts were identified from the databases search of which 446 remained after duplicates were eliminated. There was substantial interrater reliability between the three reviewers (Cohens kappa = 0.62) [38]. After screening titles and abstracts, a total of 30 full texts papers were selected and evaluated for eligibility. Thereafter, 11 studies were excluded due to inherent study design issues, incorrect population criteria and irrelevant outcomes, as reported in Online Appendix S1. Consequently, 19 SRs, based on 173 primary clinical studies, were identified, and included in this review. An overview of the selected SRs with their IDs (I‐XIX) and their characteristics is presented in Table 2. Of the final selection, 13 SRs evaluated CVD and eight studies evaluated CVE, of which one focused on cerebrovascular disease, eight on CHD, four on ACVD, one on PVD, six on stroke and three on MI.

**TABLE 2:** Overview of the characteristics of the included SRs processed for data extraction.

Author (year) CVD/CVE Risk of Bias (%) (Online Appendix S2)	Databases searched	Number of included studies/trails # involved participants base (end)	Leading mode of analysis	Original conclusions of review authors	Comments of the meta‐review authors
I: Bahekar et al. 2007 [39] CHD ROB: Moderate (63%)	○ MEDLINE‐PubMed ○ Cochrane‐ CENTRAL ○ EMBASE ○ CINAHL	15 studies ? (105324)	Meta‐analysis	A possible association between PerioD and CHD is found, as evidenced by a statistically significant increase in the incidence and prevalence of CHD among individuals with PerioD. Elevated levels of inflammatory mediators in patients with PerioD suggest their role in atherothrombogenesis leading to CHD.	○ There is considerable variation in the methods used to estimate PerioD and CHD.
II: Blaizot et al. 2009 [40] CVD ROB: Low (81%)	○ MEDLINE‐PubMed ○ Cochrane‐ CENTRAL ○ EMBASE ○ LILACS ○ Pascal ○ Biosis ○ French Public Health Database	32 studies ? (167931)	Meta‐analysis	It seems from observational studies that subjects with PerioD have higher odds and higher risk of developing CVD but the reduction in the risk of CVE associated with the treatment of PerioD remains to be investigated.	○ Three of the included studies used only tooth loss as a clinical exposure measurement related to PerioD. ○ The majority of the included studies are based on a population aged 40 years or older.
III: Dietrich et al. 2013 [41] ACVD ROB: Moderate (69%)	○ Unknown	12 studies 25,029 (?)	Descriptive analysis	The incidence of ACVD, as represented by incident CHD, cerebrovascular disease and PAD is higher in subjects with PerioD and/or worse periodontal status, compared to subjects without PerioD or with better periodontal status, independent of many established cardiovascular risk factors. However, this may not be the case in all groups of the population.	○ Some of the studies included in the analysis were based on the same study population, which raises the possibility of duplication of data, potential bias and overestimation of the results. ○ Only descriptive analysis was performed.
IV: Fagundes et al. 2019 [42] Stroke ROB: Low (96%)	○ MEDLINE‐PubMed ○ Cochrane‐ CENTRAL ○ LILACS ○ Web of Science ○ Scopus ○ OpenGrey ○ Google Scholar	11 studies ? (32213)	Meta‐analysis	This SR and meta‐analysis suggest an increased risk of stroke in patients with PerioD, especially in ischemic events. In addition, there is a strong association between both diseases. The results of the meta‐analysis showed that individuals with PerioD had a greater chance, about twice as much of suffering from some type of stroke, as to have ischemic stroke. However, these results should be evaluated with caution due to the need for well‐planned prospective studies for a more reliable conclusion.	○ There are discrepancies in the diagnostic criteria for stroke and PerioD. ○ Not all studies adjusted for confounding factors such as smoking or hypertension. This reflects the heterogeneity of the meta‐analysis.
V: Gao et al. 2021 [43] CHD ROB: Low (81%)	○ MEDLINE‐PubMed ○ Cochrane‐ CENTRAL ○ EMBASE	11 studies Only cohort studies ? (284622)	Meta‐analysis	The association between PerioD and CHD was found to be meaningful, as was the preliminary exploration of the effect of a reduced number of teeth on CHD. Although estimating cardiovascular risk is still complicated in clinical work, the number of teeth is considered a risk factor for CVD, however, not an independent one.	○ Four studies used number of teeth as their exposure variable, and 10 studies used periodontal condition as their exposure variable. While PerioD can lead to tooth loss, having fewer teeth does not necessarily mean that a person has PerioD. Studies that used the number of teeth as an exposure variable may not capture the full extent of the relationship between PerioD and CHD.
VI: Humphrey et al. 2008 [44] CHD ROB: Moderate (70%)	○ MEDLINE‐PubMed	7 studies Only cohort studies ? (227000)	Meta‐analysis Descriptive analyses	PerioD is a risk factor or marker for CHD that is independent or traditional CHD risk factors, including socio‐economic status.	○ The differences in case definition of PerioD and CHD between the included studies resulted in inconsistencies across studies and make it difficult to draw clear conclusions.
VII: Janket et al. 2003 [45] CHD Stroke ROB: Substantial (52%)	○ MEDLINE‐PubMed	9 studies Only cohort studies 107,011 (7035)	Meta‐analysis	PerioD is associated with the increased risk of development of subsequent CVD/CVE by approximately 19% in the general population. However, this summary result might still underestimate the true risk increase. Because in some studies inadequate confounding adjustment resulted in an overestimate of the RR by 12.9% and use of questionnaires resulted in an underestimate of the RR by 29.7%, the net result is still lower than the true RR.	○ The predictor variable, PerioD, was defined as gingivitis or PerioD. If gingivitis was included in the meta‐analysis is unclear.
VIII: Khader et al. 2004 [46] Cerebrovascular disease CHD ROB: Substantial (59%)	○ MEDLINE‐PubMed	11 studies ? (104710)	Meta‐analysis	Findings indicate that periodontal infection increases the risk of CHD and cerebrovascular disease. However, this meta‐analysis provided no evidence for the existence of strong associations between PerioD and CHD and cerebrovascular disease.	○ Three included studies indicated PerioD on self‐reported evaluation. ○ Three included studies are solely based on a population of men.
IX: Lafon et al. 2014 [47] Stroke ROB: Moderate (70%)	○ MEDLINE‐PubMed ○ Cochrane‐ CENTRAL ○ EMBASE ○ ISI Web of Science	9 studies Only cohort studies 138,930 (?)	Meta‐analysis	Our results are in accordance with those of previous reviews suggesting a link between stroke and PerioD. Pooled data were calculated based on results from cohort studies with various quality scores. More epidemiological and clinical studies are thus needed to clarify the relationship between these inflammatory diseases.	○ Only four of the nine included studies performed simple or complete clinical screening to indicate PerioD evaluation. The remaining studies determined this from a questionnaire or the number of teeth. ○ Three studies were included in which the patients were known to have a CVD/CVE history. ○ Four studies are based on men only. ○ The ages of the populations are not given.
X: Larvin et al. 2020 [48] CVD CHD Stroke MI ROB: Low (85%)	○ MEDLINE‐PubMed ○ Cochrane‐ CENTRAL ○ EMBASE	30 studies Only RCT and cohort studies ? (10007700)	Meta‐analysis	The results of this SR and meta‐analysis demonstrate increased risk of CVD/CVE in people with PerioD. Males and people with severe PerioD have the highest risk of developing CVD/CVE indicating possible target populations for future public health interventions and screening.	○ Since 21 studies having a critical risk of bias and 11 having a serious risk of bias, it may be suggested that the overall quality of evidence may be lower than desired. ○ The significant risk of publication bias demonstrated in the funnel plot and Egger's test indicates that the study may have included only a subset of relevant studies, potentially leading to an overestimation or underestimation of the true magnitude of the association. Nine included studies indicated PerioD on self‐reported evaluation.
XI: Leira et al. 2017 [49] Stroke ROB: Low (89%)	○ MEDLINE‐PubMed ○ EMBASE ○ Web of Science ○ Current Contents Connect	8 studies ? (14091)	Meta‐analysis Descriptive analysis	Our results for cohort studies, which are less prone to bias than case–controls studies, together with the results of high‐quality studies, show that antecedents of PerioD may be a moderate to strong risk factor of ischemic stroke.	○ No potential confounding factors that could affect the association between PerioD and ischemic stroke were analysed, which could limit the generalizability of the findings.
XII: Meregildo‐Rodriguez et al. 2022 [50] CHD ROB: Low (85%)	○ MEDLINE‐PubMed ○ EMBASE ○ Web of Science ○ Scopus ○ Science Direct ○ Google Scholar	46 studies ? (6806286)	Meta‐analysis	This study shows that PerioD would be a non‐traditional risk factor for developing ACS. The results support the hypothesis that chronic inflammation caused by PerioD is involved in the pathogenesis of ACVD and ACS. Given the high prevalence of PerioD in the population, this would have a profound impact on public health, health policy and clinical specialties, such as cardiology and neurology. Therefore, maintaining adequate periodontal health could be an effective measure to reduce the risk of ACS. However, our results should be taken with caution due to the quality of the evidence and the heterogeneity of the studies.	○ Four included studies indicated PerioD on self‐reported evaluation. ○ Seven studies are based on men only and three studies on woman only. ○ The significant risk of publication bias demonstrated in the funnel plot indicates that the study may have included only a subset of relevant studies, potentially leading to an overestimation or underestimation of the true magnitude of the association.
XIII: Orlandi et al. 2014 [51] ACVD ROB: Low (91%)	○ MEDLINE‐PubMed ○ Cochrane‐ CENTRAL ○ EMBASE ○ LILACS ○ SCI‐EXPANDED	35 studies ? (?)	Meta‐analysis Descriptive analysis	PerioD is associated with greater subclinical ACVD as assessed by increased c‐IMT and FMD.	○ A high level of heterogeneity was observed in both case–control and intervention trials, indicating differences in research protocols and clinical heterogeneity that may affect the robustness of the findings. However, the statistical heterogeneity was low.
XIV: Qin et al. 2021 [52] MI ROB: Moderate (74%)	○ MEDLINE‐PubMed ○ EMBASE ○ The Cochrane Library	10 studies Only cohort studies ? (5369235)	Meta‐analysis	PerioD is modestly associated with MI risk, especially in women.	○ After subgrouping, there was still a significant level of heterogeneity present in the meta‐analysis.
XV: Sfyroeras et al. 2012 [53] Stroke ROB: Moderate (63%)	○ MEDLINE‐PubMed	13 studies ? (123229)	Meta‐analysis	There is evidence that PerioD is associated with increased risk of stroke. However, the results of this meta‐analysis should be interpreted with caution because of the heterogeneity of the studies as well as the differences in PerioD definition.	○ Patients with ischemic and hemorrhagic stroke were both included, however separate statistical analyses were not able to be conducted. ○ Of the 13 included studies, six prospective studies compared individuals with PerioD to those without. The other studies evaluated the incidence of PerioD in individuals with CVD/CVE to those without.
XVI: Voinescu et al. 2019 [54] CHD ROB: Moderate (63%)	○ MEDLINE‐PubMed ○ Scopus ○ Science Direct ○ Google Scholar	17 studies ?	Descriptive analysis	The prevalence of PerioD in patients with ischemic cardiac disease is high, the existence of a correlation between the two pathogens being most often established in studies, although the mechanism has not been elucidated yet.	○ Description of study characteristics of the included studies are limited.
XVII: Wang et al. 2019 [55] ACVD PVD (PAD) ROB: Moderate (74%)	○ MEDLINE‐PubMed ○ Cochrane‐ CENTRAL ○ EMBASE ○ Google Scholar ○ Ovid Medline	25 studies ? (22090)	Meta‐analysis	The meta‐analysis of 25 studies identified by comprehensive SR indicates that PerioD is an independent risk factor for both CAD and LEAD.	○ Two of the included studies used only tooth loss as a clinical exposure measurement related to PerioD and one study based the PerioD exposure on self‐reported periodontal assessment. ○ Population information other than location of the studies was not given.
XVIII: Xu et al. 2017 [55] MI ROB: Low (81%)	○ MEDLINE‐PubMed ○ EMBASE ○ The Cochrane Library	22 studies ? (129630)	Meta‐analysis	The meta‐analysis yielded a statistically significant association between PerioD and MI. Subgroup analyses also confirmed the elevated risk for MI in PerioD subjects, although heterogeneity should be noted.	○ Of the 22 included studies, three cohort studies compared individuals with PerioD to those without. The other studies evaluated the periodontal health in individuals with MI history compared to those without MI history.
XIX: Zeng et al. 2015 [56] ACVD ROB: Moderate (70%)	○ MEDLINE‐PubMed ○ EMBASE	15 studies ? (17330)	Meta‐analysis	Our meta‐analysis of 15 observational studies indicates that PerioD was associated with carotid ACVD, although currently available evidence is insufficient to confirm the causal relationship of PerioD and carotid ACVD.	○ The association between PerioD and CAD may be confounded by smoking and DM, since six of the included studies did not take these factors into account while analysing the relationship between PerioD and CAD. Adjusting for these factors may weaken the observed relationship.

Abbreviations: ?, Is not reported/unknown; ACS, acute coronary syndrome; ACVD, atherosclerotic cardiovascular disease; CAD, carotid atherosclerosis; CHD, coronary heart disease; c‐IMT, carotid intima‐media thickness; CVD, cardiovascular disease; CVD/CVE, cardiovascular diseases; CVE, cardiovascular event; DM, diabetes mellitus; FMD, flow‐mediated dilatation; GRADE, grading of recommendations assessment, development, and evaluation; IHD, ischemic heart disease; LEAD, peripheral artery disease; MI, myocardial infarction; PAD, peripheral artery disease; PVD, peripheral vascular disease; PerioD, periodontitis, periodontal disease; ROB, risk of bias; RR, relative risk; SR, systematic review.

### 3.2 Assessment of Heterogeneity

The 19 SRs analysed in this synthesis demonstrated significant heterogeneity in various aspects, including the searched databases, characteristics of the original studies and their subjects, inclusion and exclusion criteria, the quality assessment scale employed, as well as the aims, methods of recording and reporting and the inclusion of meta‐analyses (Table 2).

### 3.3 Assessment of Citations

As presented in the citation matrix (Online Appendix S3), there was an overlap in the inclusion of primary clinical studies across multiple SRs, with some studies being included in more than one review.

### 3.4 Quality Assessment

The majority of the SRs evaluated in this synthesis were estimated to have a low to moderate risk of bias, as shown in Table 2 and Online Appendix S2. Two of the included reviews, VII [45] and VIII [46], were found to exhibit a substantial risk of bias. The assessment was conducted through a critical evaluation of criteria including the ‘a priori’ development and registration of a protocol, variety of searched databases or additional sources, inclusion of non‐English literature, attempts to contact authors for additional information, grading of obtained evidence and assessment of publication bias.

### 3.5 Study Outcome Results

The analysis was performed per category CVD and CVE. For a detailed analysis of the different outcome aspects, see Online Appendix S4.

#### 3.5.1 CVD

Thirteen SRs (I [39], II [40], III [41], V [43], VI [44], VII [45], VIII [46], X [48], XII [50], XIII [51], XVI [54], XVII [57] and XIX [56]) were identified evaluating the relationship between PerioD and CVD. Details and an overview of the extracted meta‐analyses data of the included SRs are shown in Tables 3A and 4A. Most of the studies further specified CVD as cerebrovascular disease, CHD, ACDV or PVD. Two SRs (II and X) performed a meta‐analyses for CVD specifically and showed a significant association of PerioD and CVD, for patients with PerioD as compared to those without. The interpretation of the OR and RR, the magnitude of the association were estimated to be negligible to small (Table 3A). The heterogeneity among the clinical studies in the meta‐analyses is estimated to range from potentially not significant to considerable (Table 3A).

**TABLE 3:** Overview of data extraction of the included SRs and for subgroup analysis.

Source				Outcome			Heterogeneity	
Disease or event	SR	No. studies in meta‐analysis (variable)	No. patients in meta‐analysis	Effect model (Fixed/Random): OR/RR*	95% CI	*p* value	I^2^, Q*, X^2^**, Ri***	*p* value
**CVD**	II [40]	32 (total)	167,931	NR	NR	NR	NR	NR
		7 (cohort)	147,821	Fixed: 1.34*	1.27–1.42	**< 0.0001**	5.6%	0.39
		22 (cs & cc)	20,110	Random: 2.35	1.87–2.96	**< 0.0001**	49.9%	0.0044
	X [48]	30 (total)	10,007,700	NR	NR	NR	NR	NR
		26 (CVD)	9,491,880	Random: 1.20*	1.14–1.28	**sig**	98.1%	0.00
**Cerebrovascular disease**	VIII [46]	6 (total)	36,915	Random: 1.13*	1.01–1.27	**0.032**	?	?
		4 (cohort)	33,744	Random: 1.11*	0.98–1.25	0.106	?	?
**CHD**	I [39]	15 (total)	105,324	NR	NR	NR	NR	NR
		5 (cohort)	86,092	Fixed: 1.14*	1.07–1.21	**< 0.001**	homog	not sig
		5 (cs)	17,809	Fixed: 1.59	1.33–1.91	**< 0.001**	homog	not sign
		5 (cc)	1423	Fixed: 2.22	1.59–3.12	**< 0.001**	homog	not sign
	V [43]	11 (total)	284,622	NR	NR	NR	NR	NR
		10 (PerioD vs. non–PerioD)	175,795	Fixed: 1.18	1.10–1.26	**sig**	11.7%	0.330
	VI [44]	6 (total)	33,163	Random: 1.24*	1.01–1.51	**sig**	13.03*	0.048
	VII [45]	9 (total, CHD)	7035	Random: 1.19*	1.08–1.32	**0.000**	23.624*	0.003
	VIII [46]	8 (total)	94,096	Random 1.15*	1.06–1.25	**0.001**	homog**	0.472
		6 (cohort)	85,693	Random: 1.14*	1.04–1.25	**0.004**	?	?
		3 (cohort, fatal)	9777	Random: 1.20*	0.90–1.60	0.205	?	?
	X [48]	13 (CHD)	3,332,392	Random: 1.14*	1.08–1.21	**sig**	68.4%	0.00
	XII [50]	46 (total)	6,806,286	Random: 1.35	1.25–1.45	**< 0.00001**	86%	< 0.00001
		17 (cohort)	6,787,657	Random: 1.13	1.05–1.21	**0.0010**	81%	< 0.00001
		4 (cs)	9056	Random: 1.67	0.79–3.50	0.18	46%	0.13
		25 (cc)	9573	Random: 2.62	2.05–3.35	**< 0.00001**	86%	< 0.00001
	XVI [54]	NA	NA	NA	NA	NA	NA	NA
**ACVD**	III [41]	NA	NA	NA	NA	NA	NA	NA
	XIII [15]	22 (total)	6034	NR	NR	NR	NR	NR
		16 (c–IMT)	5452	NA	NA	**< 0.0001**	0.0%	0.922
		6 (FMD)	582	NA	NA	**< 0.001**	80.1%	0.000
	XVII	9 (LEAD)	4468	Random: 3.00	2.23–4.04	**< 0.001**	0.0%	0.563
	XIX [56]	15 (total)	17,330	Random: 1.27	1.14–1.41	**< 0.01**	78.9%	< 0.01
		10 (PerioD vs. non–PerioD)	3961	Random: 1.75	1.33–2.30	**< 0.01**	74.64%	< 0.01
**PVD (PAD)**	XVII [55]	25 (total)	22,090	Random: 1.60	1.41–1.82	**< 0.001**	80.5%	0.000
		16 (CAD)	18,902	Random: 1.39	1.24–1.56	**< 0.001**	79.4%	0.000
**CVE**								
**Stroke**	IV [42]	11 (total)	32,213	NR	NR	NR	NR	NR
		3 (cohort)	28,900	Random: 1.88*	1.55–2.28	**< 0.00001**	0%	0.59
		7 (cc)	1513	Random: 2.31	1.39–3.84	**0.001**	77%	0.0003
		4 (cc, ischemic stroke)	1015	Random: 2.72	2.00–3.71	**< 0.00001**	4%	0.37
	VII [45]	2 (stroke)	1010	Random: 2.85*	1.78–4.56	**0.000**	?	?
		5 (fatal)	4733	Random: 1.54*	1.10–2.17	**0.012**	?	?
	X [48]	18 (stroke)	3,651,223	Random: 1.24*	1.12–1.38	**sig**	95.5%	0.00
	IX [47]	9 (total)	138,930	NR	NR	NR	NR	NR
		4 (PerioD outcomes)	27,730	Random: 1.63*	1.25–2.00	**sig**	homog	NR
	XI [49]	8 (total)	14,091	Fixed: 1.07*	1.03–1.11	**sig**	1.0***	0.00001
				Random: 2.88*	1.53–5.41			
		3 (cohort)	12,246	Fixed: 2.52*	1.77–3.58	**sig**	1.0***	0.50
				Random: 2.52*	1.77–3.58			
		5 (cc)	1845	Fixed: 1.06*	1.02–1.10 1.10–8.43	**sig**	0.0***	0.00001
				Random: 3.04*				
		4 (high‐quality studies)	1750	Fixed: 4.83*	3.13–7.46	**sig**	0.0***	0.47
				Random: 4.83*	3.13–7.46			
		4 (low‐quality studies)	12,341	Fixed: 1.06*	1.02–1.10	**sig**	1.0***	0.00001
				Random: 2.02*	1.06–3.85			
	XV [53]	13 (total)	123,229	NR	NR	NR	NR	NR
		6 (prospective studies)	105,247	Random: 1.47*	1.13–1.92	**0.0036**	heterog	sig
**MI**	X [48]	6 (MI)	771,925	Random: 1.12*	0.96–1.30	not sig	82.9%	0.00
	XVIII [55]	22 (total)	129,630	NR	NR	NR	NR	NR
		4 (cohort)	47,819	Random: 1.18	0.98–1.42	not sig	64.8%	0.036
	XIV [52]	10 (total)	5,369,235	Random: 1.13	1.04–1.21	**0.004**	78%	0.000
		5 (high‐quality studies)	5,270,520	Random: 1.20	1.03–1.37	**0.019**	86.5%	0.000
		5 (moderate‐quality studies)	98,715	Random: 1.09	0.94–1.24	0.254	64.1%	0.025

Subgroup analysis								
**Gender**	III [41]	NA	NA	NA	NA	NA	NA	NA
	VI [44]	? (combined)	?	Random: 1.31*	1.08–1.59	not sig	?	?
		? (women)	?	Random: 1.59*	1.28–1.96	?	?	?
		? (men)	?	Random: 1.23*	0.92–1.64	?	?	?
	X [48]	16 (combined)	6,467,175	Random: 1.04*	0.92–1.17	not sig	90.7%	0.00
		6 (women)	1,691,927	Random: 1.11*	1.02–1.22	?	85.8%	0.00
		14 (men)	4,775,248	Random: 1.16*	1.08–1.25	?	96.6%	0.00
	XII [50]	37 (combined)	6,714,856	Random: 1.35	1.24–1.45	0.67	86%	< 0.00001
		2 (woman)	40,056	Random: 1.96	0.62–6.17	0.25	87%	0.005
		7 (men)	51,374	Random: 1.48	1.11–1.97	0.007	61%	0.02
	XIV [52]	NR (combined)	NR	NR	NR	NR	NR	NR
		1 (women)	39,863	Random: 1.39	1.17–1.65	**0.000**	NA	NA
		3 (men)	498,053	Random: 1.05	0.89–1.24	0.560	18.0%	0.300
**Age**	II [40]	? (cohort, mean age)	?	?	?	0.06	?	?
		? (cs, cc, mean age)	?	?	?	0.11	?	?
	III [41]	NA	NA	NA	NA	NA	NA	NA
	VII [45]	6 (≤ 65 years)	2890	Random: 1.44*	1.20–1.73	**0.000**	23.624*	0.003
**PerioD severity**	III [41]	NA	NA	NA	NA	NA	NA	NA
	X [48]	18 (combined)	7,016,015	Random: 1.20*	1.15–1.25	?	90.5%	0.00
		18 (severe vs. mild)	4,724,176	Random: 1.11*	1.00–1.22	**sig**	?	?
		18 (severe)	2,165,275	Random: 1.25*	1.15–1.35	?	71.6%	0.00
		15 (moderate)	2,291,839	Random: 1.23*	1.14–1.32	?	70.6%	0.00
		12 (mild)	2,558,901	Random: 1.09*	1.05–1.14	?	80.0%	0.00
	XVI [54]	NA	NA	NA	NA	NA	NA	NA
	XIX [56]	NR (combined)	NR	NR	NR	NR	NR	NR
		5 (severe)	13,369	Fixed: 1.14	1.06–1.23	**< 0.01**	47.16%	0.11
		4 (moderate)	13,166	Fixed: 1.1	1.04–1.16	**< 0.01**	0%	0.9
**Smoking status**	III [41]	NA	NA	NA	NA	NA	NA	NA
	XVI [54]	NA	NA	NA	NA	NA	NA	NA
**Study region**	II [40]	? (cohort, North America)	?	Fixed: 1.50*	1.34–1.68	0.08	?	?
		? (cohort, Scandinavia)	?	Fixed: 1.30*	1.17–1.44			
		? (cohort, China)	?	Fixed: 1.28*	1.17–1.40			
		? (cs, cc, Europe)	?	Random: 3.95	2.54–6.15	0.08	?	?
		? (cs, cc, South America)	?	Random: 2.34	1.48–3.70			
		? (cs, cc, North America)	?	Random: 2.02	1.49–2.73			
		? (cs, cc, Scandinavia)	?	Random: 2.00	1.27–3.15			
	X [48]	30 (combined)	10,007,700	Random: 1.20*	1.14–1.26	?	97.3%	0.00
		4 (Europe)	134,035	Random: 1.36*	1.20–1.54	?	71.3%	0.00
		16 (North America)	835,583	Random: 1.15*	1.09–1.26	?	80.3%	0.00
		10 (Asia/ Australia)	9,038,082	Random: 1.20*	1.11–1.30	?	98.1%	0.00
		4 (Europe vs. Asia/ Australia)	9,172,117	Random: 1.18*	1.03–1.35	?	?	?
		16 (Europe vs. North America)	969,618	Random: 1.03*	0.93–1.13	?	?	?
	XII [50]	46 (combined)	6,806,286	Random: 1.35	1.25–1.45	**< 0.00001**.	86%	< 0.00001
		13 (North America)	134,107	Random: 1.30	1.16–1.46	**< 0.00001**	67%	0.00003
		4 (South America)	920	Random: 4.43	2.39–8.23	**< 0.00001**	0%	0.95
		20 (Europe)	126,163	Random: 1.92	1.59–2.31	**< 0.00001**	84%	< 0.00001
		9 (Asia)	6,545,096	Random: 1.09	0.96–1.25	0.20	86%	< 0.00001

Abbreviations: ?, Is not reported/unknown; ACVD, Atherosclerotic cardiovascular disease; CAD, Carotid atherosclerosis; CHD, Coronary heart disease; Cc, case–control study; c‐IMT, Carotid intima‐media thickness; Cs, cross‐sectional study; CVD, Cardiovascular disease; CVE, Cardiovascular event; FMD, Flow‐mediated dilatation; Heterog, Heterogeneous; Homog, Homogeneous; LEAD, Peripheral artery disease; NA, Not appliable; NR, Not reported; MI, Myocardial infarction; OR, Odds Ratio; PAD, Peripheral artery disease; PVD, Peripheral vascular disease; PerioD, Periodontitis, Periodontal disease; Q*, Cochran's *Q* test for heterogeneity; Ri***, DerSimonian and Laird *Q* test for heterogeneity; RR, Relative risk; RR*, Relative Risk; Sig; significant; SR, Systematic review; X^2^**, Chi‐square test for heterogeneity.

*Note:*

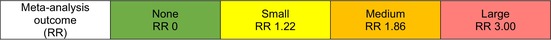
 As a guideline, the magnitude of the association of the RR was interpreted using a normal standard deviation. Effect sizes of 1.22, 1.86 and 3.00 were considered indicative of small, medium and large magnitudes, respectively [29].
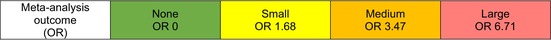
As a guideline, the magnitude of the association of the OR was interpreted using a normal standard deviation. Effect sizes of 1.68, 3.47 and 6.71 were considered indicative of small, medium and large magnitudes, respectively [30].
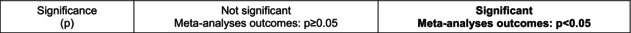
Meta‐analyses outcomes resulting in a *p* < 0.05 was considered to be statistically significant. 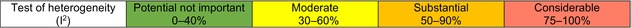

As a guideline, to assess the potential magnitude of inconsistency between studies, an I^2^ statistic of 0%–40% may represent unimportant levels of heterogeneity, 30%–60% may represent moderate heterogeneity, 50%–90% may represent substantial heterogeneity. An I^2^ statistic of greater than 75% was interpreted to indicate considerable heterogeneity [31].

**TABLE 4:** Summary of the magnitude of the association of the extracted RRs and ORs for CVD; RRs and ORs for CVE.

(A)CVD						
SR	Total number of included studies	Number of performed meta‐analysis	Magnitude of the association of the extracted RRs [29] and ORs [30]			
			None	Small	Medium	Large
I [39]	15	3	2/3	1/3		
II [40]	32	2		2/2		
III [41]	12	0	NA			
V [43]	11	1	1/1			
VI [44]	7	1	1/1			
VII [45]	9	1	1/1			
VIII [46]	11	5	5/5			
X [48]	30	2	2/2			
XII [50]	46	4	3/4	1/4		
XIII [51]	35	3	3/3			
XVI [54]	17	0	NA			
XVII [57]	25	3	2/3	1/3		
XIX [56]	15	2	1/2	1/2		
Summary		27	8.6/11= 78%	2.4/11= 22%	0/11= 0%	0/11= 0%
Overall		Of the 11 SRs that performed a meta‐analysis, the majority (78%) of the values of RRs and ORs are considered to show a negligible magnitude of the association between PerioD and CVD.				

(B) CVE						
IV [42]	11	3		2/3	1/3	
VII [45]	9	2		1/2	1/2	
IX [47]	9	1		1/1		
X [48]	30	2	1/2	1/2		
XI [49]	8	10	3/10		4/10	3/10
XIV [52]	10	3	3/3			
XV [53]	13	1		1/1		
XVIII [55]	22	1	1/1			
Summary		23	2.8/8= 35%	3.7/8= 46%	1.2/8= 15%	0.3/8= 4%
Overall		Of the 8 SRs, that performed a meta‐analysis, the majority (46%) of the values of RRs and ORs are considered to show a small magnitude of the association between PerioD and CVE.				

Abbreviations: CVE, cardiovascular event; NA, not appliable (no meta‐analysis available); OR, odds ratio; PerioD, periodontitis, periodontal disease; RR, relative risk; SR, systematic review.

##### 3.5.1.1 Cerebrovascular Disease

One SR (VIII [46]) was identified which evaluated the association between PerioD and cerebrovascular disease. The results of the performed meta‐analyses showed a significantly increased risk of cerebrovascular disease for patients with PerioD in comparison to individuals without PerioD. As shown in Table 3A, the interpretation of the values of the extracted RR indicates that the magnitude of the association can be considered as negligible. The statistical heterogeneity associated with the meta‐analysis is not described in this SR (Table 3A).

##### 3.5.1.2 CHD

Eight SRs (I [39], V [43], VI [44], VII [45], VIII [46], X [48], XII [50] and XVI [54]) were identified, examining the association between PerioD and CHD. All these reviews revealed a significant relationship, indicating an elevated risk of CHD for patients with PerioD compared to those without. Interpretation of the ORs and RRs is detailed in Table 3A, suggesting that the magnitude of the association can be estimated to range from negligible to small. Based on the findings of the meta‐analyses, it appears that the statistical heterogeneity among the included clinical studies ranges from potentially not important to considerable (Table 3A).

##### 3.5.1.3 ACVD

Four SRs (III [41], XIII [51], XVII [57] and XIX [56]) were identified which evaluated the association of PerioD and ACVD. All studies showed a significant relationship, with an increased risk for patients with PerioD compared to those without. However, the range of the ORs implies that the value of the association is estimated to range from negligible to small. The meta‐analyses indicate that the heterogeneity among the clinical studies ranges from potentially not important to considerable (Table 3A).

##### 3.5.1.4 PVD

A single SR (XVII [57]) was identified assessing the association between PerioD and PAD of which the results indicate a significantly heightened risk for patients with PerioD in developing PAD. However, on interpreting the OR, the actual magnitude of the association is estimated to be negligible (Table 3A). Based on the meta‐analyses, the statistical heterogeneity was also considerable among the included studies (Table 3A).

#### 3.5.2 CVE

Eight SRs (IV [42], VII [45], IX [47], X [48], XI [53], XIV [52], XV [53] and XVIII [40]) were identified evaluating the association between PerioD and CVE. Details and an overview of the extracted data of the meta‐analyses of the included SRs are shown in Table 3A and Table 4B. All studies further specified CVE as MI or stroke.

##### 3.5.2.1 Stroke

Six SRs (IV [42], VII [45], IX [47], X [48], XI [49] and XV [53]) were identified which evaluated the association of PerioD and stroke. All studies showed a significant relationship, with an increased risk for patients with PerioD compared to those without. The interpretation of the extracted ORs and RRs (for details, see Table 3A) shows that the magnitude of the association ranges from none to large. As emerging from the meta‐analyses, the heterogeneity among the included clinical studies ranges from potentially not important to considerable (Table 3A).

##### 3.5.2.2 MI

Three SRs (X [48], XIV [52] and XVIII [55]) were identified which evaluated the relationship between PerioD and MI of which the results suggest an association between PerioD and the risk of MI. However, the interpretation of the association indicated that the magnitude of the ORs could be considered as negligible (Table 3A). The statistical heterogeneity among the clinical studies ranges from substantial to considerable (Table 3A).

#### 3.5.3 Subgroup Analysis

Nine SRs (II [40], III [41], VI [44], VII [45], X [48], XII [50], XIV [52], XVI [54] and XIX [56]) performed sub‐analyses evaluating the association of PerioD and CVD/CVE relative to parameters for gender, age, smoking status, PerioD severity or study region. Details and an overview of the extracted data of the meta‐analyses for the subgroup analysis in the included SRs are shown in Table 3B.

##### 3.5.3.1 Gender

Five SRs (III [41], VI [44], X [48], XII [50] and XIV [52]) evaluated the association of PerioD and CVD/CVE in relation to gender. Three SRs suggested a higher risk of CVD/CVE in women with PerioD (VI, XII and XIX) while a greater risk was observed in men according to one SR (III). The observed differences between genders were not statistically significant and the estimation of the magnitude of the association based on ORs and RRs values ranges from none to small (Table 3B). As indicated by the meta‐analyses, the heterogeneity among the included clinical studies is in the range from potentially not important to considerable (Table 3B). Consequently, the association between PerioD and CVD/CVE in relation to gender remains inconclusive.

##### 3.5.3.2 Age

Three SRs (II [40], III [41] and VII [45]) evaluated the association of PerioD and CVD/CVE relative to age. The findings suggest that age may play a role in the association between PerioD and CVD/CVE, with younger age groups (≤ 65 years old) experiencing a potentially higher risk. The observed OR from one meta‐analysis (VII) implies that the estimation of the magnitude of the association is considered small (Table 3B). No information was provided on statistical heterogeneity in the meta‐analyses.

##### 3.5.3.3 PerioD Severity

Four SRs (III [41], X [48], XVI [54] and XIX [56]) evaluated the association of PerioD and CVD/CVE in relation to PerioD severity. The findings suggest that PerioD severity may play a role in the association between PerioD and CVD/CVE, with individuals with severe PerioD experiencing a significantly higher risk. Interpreting the ORs and RRs, the magnitude of the association was estimated to range from negligible to small (Table 3B). The statistical heterogeneity emerging from the meta‐analyses ranges from potentially not important to considerable among the included clinical studies (Table 3B).

##### 3.5.3.4 Smoking Status

Two SRs (III [41] and XVI [54]) evaluated the association of PerioD and CVD/CVE relative to the smoking status. The association between PerioD and CVD/CVE parameters in relation to smoking status remains inconclusive as conclusions of both SRs were based on limited evidence from a single clinical study. Assessment of heterogeneity with one included study was not applicable.

##### 3.5.3.5 Study Region

Three SRs (II [40], X [48] and XII [50]) evaluated the association of PerioD and CVD/CVE in relation to the region where the clinical study was performed. The findings suggest that this may play a role in the association between PerioD and CVD/CVE, where only one SR (XII) found this difference to be statistically significant. Additionally, interpreting the extracted ORs and RRs shows that the magnitude of the association ranges from none to medium (Table 3B). Meta‐analyses showed that among the included clinical studies, the heterogeneity was in a range from potentially not important to considerable (Table 3B).

### 3.6 Assessment of Causality

The application of the Bradford Hill criteria [18] as summarised in Table 5 and Online Appendix S5 shows the detailed analysis. Three out of the nine criteria, namely consistency, biological gradient and plausibility, can be satisfied. Regarding the causality of the association between PerioD and CVD and CVE, it is important to recognise that definitive establishment of causality cannot be inferred solely from the findings of the current synthesis.

**TABLE 5:** Overview of the Bradford Hill criteria [18] for causality [58].

Criteria	Meaning	CVD		CVE	
		Yes	No	Yes	No
Strength of association (Table 3)	A strong association is more likely to have a causal component than a modest association. The strength of the association is determined by the types of existing studies. The highest‐level studies from the evidence pyramid would represent the strongest associations (i.e., RCTs and SRs with meta‐analyses). Results from these studies must demonstrate an **OR or RR of at least 2.0** or above to be meaningful. Anything between 1 and 2 is weak while > 2 is moderate and > 4 is considered strong.		x		x
Consistency	A relationship is repeatedly observed in all available studies.	x		x	
Specificity	A factor influences specifically a particular outcome or population. The more specific an association between a factor and an effect, the greater the probability that it is causal.		x		x
Temporality	The cause must precede the outcome it is assumed to affect (e.g., smoking before the appearance of lung cancer). Outcome measured over time (longitudinal study).		x		x
Biological gradient (dose–response)	The outcome increases monotonically with increasing dose of exposure or according to a function predicted by a substantive theory (e.g., the more cigarettes one smokes, the greater the chance of the cancer occurring).	x		x	
Plausibility	The observed association can be plausibly explained by substantive matter (i.e., biologically possible).	x		x	
Coherence	A causal conclusion should not fundamentally contradict present substantive knowledge. Studies must not contradict each other.		x		x
Experiment	Causation is more likely if evidence is based on randomised experiments or a systematic review of randomised experiments. However, RCTs may not be ethically possible and thus prospective rather than experimental studies, such as cohort studies, may be the highest level of evidence available.		x		x
Analogy	For analogous exposures and outcomes, an effect has already been shown (e.g., effect first demonstrated on animals or an effect previously occurring on humans such as the effects of thalidomide on a fetus during pregnancy).		x		x

Abbreviations: CVD, cardiovascular disease; CVE, cardiovascular event; OR, odds ratio; RCT, randomised controlled trial; RR, relative risk; SR, systematic review.

### 3.7 Evidence Profile

The strength of the body of evidence based on GRADE [35, 36] is summarised in Table 6. With respect to the relationship of PerioD and parameters of CVD/CVE, there is moderate certainty that the magnitude of this association can be estimated as negligible to small, and causality, according to Bradford Hill criteria, remains undefined. The list of abbreviations can be found in Online Appendix S6.

**TABLE 6:** Estimated evidence profile (GRADE) [35] for the effect of PerioD on various cardiovascular parameters.

GRADE	CVD	CVE
Study designs	SRs (*N* = 13)	SRs (*N* = 8)
Reporting and methodological estimated potential risk of bias (Table 5)	Low to substantial	Low to substantial
Consistency	Rather consistent	Rather consistent
Heterogeneity (Table 2)	Rather heterogenous	Rather heterogenous
Directness	Rather indirect	Rather direct
Precision	Rather precise	Rather precise
Publication bias	Possible	Possible
Magnitude of the effect (Table 4) [29, 30]	Negligible	Negligible to Small
Certainty	Moderate	Moderate
Bradford Hill criteria [18]	Uncertain causal relationship	Uncertain causal relationship
Body of evidence	Regarding the association between PerioD and CVD/CVE, there is a moderate level of certainty that the magnitude is negligible to small.	

Abbreviations: CVD, cardiovascular disease; CVD/CVE, cardiovascular diseases; CVE, cardiovascular event; PerioD, periodontitis, periodontal disease; SR, systematic review.

## 4 Discussion

### 4.1 Summary of the Findings

This synthesis aimed to summarise the existent dental and medical evidence from SRs concerning the association of PerioD and parameters of CVD/CVE. Specifically, SRs were included due to their comprehensive nature and ability to provide a stronger body of evidence compared to individual clinical studies. The analysis of the 19 SRs included in this synthesis consistently demonstrated an increased risk of CVD and CVE in patients with PerioD compared to individuals without PerioD. Nevertheless, the magnitude of the association was in general estimated to be negligible to small (see Table 4A,B). Statistical significance and magnitude of the association varied and factors including gender, age, the severity of PerioD, smoking status and geographic location may affect the link between the PerioD and CVD/CVE. However, it is important to note that there was considerable heterogeneity across the included clinical studies and directly comparing the SRs is challenging due to variations in the used approaches to assess PerioD and CVD/CVE among the included clinical studies. Besides the relatively negligible to small association between PerioD and CVD/CVE, the findings should also be interpreted with caution considering the limitations and potential biases in the data that emerged from the included studies.

### 4.2 The Association Between PerioD and CVD

Previous papers have suggested a link between the two conditions due to their common causal components, such as lifestyle factors and smoking [59, 60, 61]. It is known that PerioD causes chronic inflammation demonstrated with elevated plasma cytokines in gingiva and gingival fluid which inflammatory markers are also shown to be elevated systemically [62]. The potential causal relationship between the two conditions could be explained by various mechanisms, including endothelial dysfunction, direct damage caused by periodontal pathogens, inflammation resulting from microbial byproducts and immune reactions triggered by bacterial antigens. These factors collectively contribute to the development and progression of both conditions [62].

This synthesis shows that the overall findings from multiple clinical studies suggest a significant association between PerioD and various CVD outcomes. The overall available evidence suggests a statistically significant link between PerioD and CVD, particularly for CHD and ACVD. However, when interpreting the RRs and ORs, the magnitude of the association of PerioD and CVD was estimated to range from negligible to small (Tables 3A and 4).

The heterogeneity among the studies should also be considered as this may have an impact on the interpretation of the findings. The variations in study design, measurement tools and adjustment for confounding factors contribute to the heterogeneity observed. Moreover, some studies have suggested potential biases and confounding factors that could affect the accuracy of the findings.

### 4.3 The Association Between PerioD and CVE

In accordance with a recent consensus report by Sanz et al. [63], which stated that patients with PerioD have an elevated risk for both stroke and MI, this synthesis shows evidence of a higher risk of stroke in patients with PerioD compared to those without. Sensitivity analyses and subgroup analyses were conducted in some studies, revealing that high‐quality studies tended to show a stronger association between PerioD and stroke, while low‐quality studies showed weaker but still significant associations (XI [49]). These differences were also notable when interpreting the extracted ORs and RRs, showing that the magnitude of the association could be interpreted to range from none to medium for most meta‐analyses, yet large for meta‐analyses outcomes of high‐quality studies (Tables 3A and 4).

A recent publication in the Journal of the American Dental Association [64] revealed an elevated risk of hospitalisation due to MI in patients with PerioD. While this synthesis similarly indicates a higher risk for MI, the findings lack consistent statistical significance. The meta‐analyses showed conflicting results, with observed ORs ranging from 1.09 to 1.20 (XIV [52] and XVIII [55], Table 3A) for MI in patients with PerioD, suggesting a negligible to marginal magnitude of association. Varying quality among analysed SRs, differences in study design, population characteristics and data analysis contribute to heterogeneity in the studies. In summary, a consistent and significant association between PerioD and an increased risk of stroke is observed, whereas the evidence for a link between PerioD and MI is less conclusive. Nevertheless, for both CVE, it is important to consider that mostly a small magnitude of the association between PerioD and CVE emerged (Tables 3A and 4B).

### 4.4 Individual Factors That May Contribute to the Association of PerioD on Parameters of CVD

Several of the included SRs examined gender, age, PerioD severity, smoking status and/or study region in relation to the potential association of PerioD and CVD/CVE.

Previous studies have indicated potential variations in systemic pathologies between males and females due to hormonal gender differences [65, 66]. Still, the findings regarding to the influence of gender on the association between PerioD and CVD/CVE are inconsistent. Some SRs suggest a stronger association in men (III [41]), while others indicate a higher risk in women (VI [44], XII [50] and XIX [56], Table 3B). But the differences in risk between genders were generally not found to be statistically significant and the influence of the magnitude of the association was estimated to range from none to small.

Based on the findings of three SRs (II [40], III [41] and VII [45], Table 3B), age was estimated to have an impact on the association between PerioD and CVD/CVE. Younger individuals appear to have stronger associations with CHD, while there is no clear association in individuals of 65 years and above (III [41] and VII [40], Table 3B). Additionally, the risk of CHD is further increased in individuals aged 65 years or younger with PerioD. A similar observation is reported in a recently published critical appraisal [67], emphasising the age‐dependent association between ACVD and PerioD. Generally, in men older than 60 years, no discernible association is identified between PerioD and the incidence of CVE [67].

In accordance with the consensus report of Sanz et al. [63], this synthesis shows that the severity of PerioD is consistently associated with a higher risk of CVD and CVE, showing an association between PerioD and CVD/CVE with an estimated range of negligible to small (III [41], X [48], XVI [54] and XIX [56], Table 3B). However, it should be noted that there was heterogeneity between the studies in PerioD severity description and only four SRs specifically analysed the magnitude of the association of PerioD severity on the relationship between PerioD and CVD/CVE. Therefore, the observed associations in this synthesis should be interpreted with caution.

The findings regarding the influence of smoking on the association between PerioD and CVD/CVE are conflicting. One SR (III [41]) found an association suggesting that the relationship between PerioD and CVD/CVE may be stronger in non‐smokers compared to those who have smoked before. Contrary, one other SR (XVI [54], Table 3B) stated that there is an increased risk of CVD and CVE associated with PerioD among smokers. However, it is important to note that these findings are based on limited evidence since both SRs mentioned that their findings relied on a single clinical study to support their conclusions regarding the association between smoking status, PerioD and CVD/CVE [68, 69]. Additionally, it is important to consider the broader context and acknowledge the existing literature that highlights the relationship between smoking, PerioD and CVD/CVE. There is evidence that smoking causes a persistent inflammatory response, which affects fibrocyte function and tissue healing. This response is linked as a risk factor to both PerioD and CVD/CVE [70]. Given these associations, smokers with PerioD should be aware of the potential increased risk of CVD/CVE and take active measures to control their cardiovascular risk factors, including smoking cessation [63].

The emerging evidence for this synthesis suggests a negligible to medium influence of the geographic area of origin on the observed risk of CVD/CVE in patients with PerioD (II [40] and X [48], Table 3B). Subgroup analyses showed higher ORs for CVD/CVE in people with PerioD from Europe, South America, North America and Scandinavian countries compared to other regions (II [40], Table 3B). Specifically, comparing European studies to Asia and Australia, a higher incident risk of CVD/CVE was found for studies conducted in Europe (X [48], Table 3B). However, only one SR found these differences to be statistically significant (XII [50], Table 3B).

### 4.5 Interpretation of the Magnitude of the Association

Table 4A,B illustrate a concise summary and interpretation of the results obtained from Table 3A. In this context, an effort was made to synthesise the dispersion of values for both RRs and ORs, ultimately showing the overall most frequently reported magnitude of the association. Yet, it is important to critically assess the interpretative value of this approach and acknowledge that the interpretation of Table 4A,B may have limitations. While it offers a simplified overview of the dispersion and the overall most frequently reported magnitude of the association of RRs and ORs, it should be viewed as a preliminary step in synthesising the data emerging from the included SRs.

As shown in Tables 3A and 4A, the magnitude of the association between PerioD and CVD was found to range from negligible to small. This could be attributed to the presence of shared risk factors. It is known that both conditions share common causal components such as smoking, DM and genetic predisposition [71, 72]. As a result, the observed association may be attenuated or diluted due to the overlapping influence of these shared risk factors. Therefore, it is important to consider these shared risk factors when evaluating the association between PerioD and CVD to more accurately assess the potential impact of PerioD on cardiovascular health. On the other hand, a noteworthy consideration is that the meta‐analyses with outcomes that ranged for small to almost medium consist of relatively smaller sample sizes (Table 3A). This observation raises an important point regarding the impact of sample size estimation on the magnitude of the association in meta‐analyses. In those studies that included smaller sample sizes, the precision of the outcomes tends to be lower [73].

The magnitude of the association that emerged from the meta‐analyses with respect to the association of PerioD and CVE ranged from negligible to large (Tables 3A and 4B). The findings presented indicate that there is a notable difference in the interpretable magnitude of the association of the ORs and RRs especially for stroke across the different SRs (Table 3A). Specifically, the meta‐analyses of high‐quality studies and case–control studies conducted by SR XI [49] showed a large magnitude of the association, suggesting a strong association between PerioD and stroke. However, it is worth noting that 2 years later, another SR was conducted (IV [42]), which included also two of the primary clinical studies previously examined by SR XI [49] (Online Appendix S3). Despite these two case–control studies being considered high‐quality studies by the reviewers of SR XI [49], the magnitude of the association calculated in the meta‐analysis in SR IV [42] decreased to a medium level after the inclusion of more recent clinical studies (Table 3A). This observation suggests that the genuine magnitude of the association between the variables is likely to be lower than initially estimated by SR XI [49]. The inclusion of newer studies, which may have contributed additional data and insights, led to a reduction in the magnitude of the association. This emphasises the importance of considering the cumulative evidence and the potential impact of new studies on values emerging from the meta‐analyses. Moreover, it is worth noting that there is notable overlap among the primary clinical studies included in the SRs, with RRs and ORs indicating values ranging from small to large magnitude (Online Appendix S2). Additionally, except for SR X [48], the meta‐analyses with an estimated small to large association consist of relatively smaller sample sizes (Table 4B) [73].

Furthermore, SR IV [42] primarily analyze the association through cohort and case–control studies. Therefore, the reliability of these results is called into question by the review authors due to substantial heterogeneity among the included SRs. It is essential to recognise and acknowledge this heterogeneity, underscoring the importance of approaching the interpretation with caution and considering potential limitations, particularly the impact of variations between the included studies.

### 4.6 Citation Matrix in the Context

When examining multiple SRs, it is important to review the primary studies and compare them to identify any potential overlap [28, 74]. For this synthesis, a citation matrix was prepared to create a comprehensive overview of primary clinical studies as included in the underlying SRs [28]. This helps to ensure that the conclusions drawn are based on a diverse range of studies. The primary studies mostly included in multiple SRs are Destefano et al. [75], Beck et al. [59], Joshipure et al. [76], Wu et al. [77], Morrisson et al. [78] and Howell et al. [79], with publication dates between 1993 and 2001 (Online Appendix S3).

With an overlap of seven to 10 primary study inclusions in SRs, it may be suggested that these studies provide valuable and reliable evidence in investigating the possible link between PerioD and CVD/CVE (Online Appendix S3). Those studies included a substantial number of participants and were of considerable epidemiological scale. Additionally, the authors found similar outcomes showing an increased risk of CVD/CVE for patients with PerioD which strengthens the confidence in the findings and increases the robustness of the evidence. Consistency across multiple reviews adds weight to the results and enhances the reliability of the conclusions drawn from the collective body of evidence [18].

Conversely, the overlap in primary studies has the potential to artificially inflate the accuracy of the analysis by overestimating the sample size and events. Including the same study multiple times in an analysis could influence the results and unwarranted weight [28, 80]. Overlapping reviews could also indicate excessive research efforts [80]. To ensure precision and reliability in SRs, addressing the overlap issue is imperative. Nevertheless, a standardised methodological approach to manage the inclusion of clinical studies across multiple SRs lacks consensus [28].

### 4.7 Appraisal of the Evidence

The present synthesis is a comprehensive evaluation of existing SRs, aiming to include all available reviews and conducting a thorough assessment of each included review [81]. The rigorous methodology, incorporating established checklists such as PRISMA [21, 22], AMSTAR [23], MOOSE [20] and the JBI [19] guidelines facilitated the assessment of the quality and validity of the included SRs. The PRISMA checklist [21] guided the SR process, promoting transparency and completeness in reporting the search strategy, study selection and data extraction. The utilisation of the AMSTAR checklist [23] allowed for a comprehensive assessment of the methodological quality of the included SRs, encompassing aspects such as study design, bias evaluation and statistical analysis. Additionally, the MOOSE [20] and JBI checklist [19] played a central position in critically appraising the quality of evidence and further strengthening the review's reliability (Online Appendices S7 and S8). Through strict adherence to these standardised checklists, this synthesis minimised potential biases, enhancing the credibility and robustness of the synthesised evidence.

The results of the PRISMA [21, 22] and AMSTAR [23] modified checklists are displayed in Table 2, Online Appendices S2 and S8, indicate that the assessed risk of bias is predominantly categorised as low to moderate. Two SRs (VII [45] and VIII [46]) included in this review were identified to have a substantial risk of bias based on critical evaluation criteria. Notably, these reviews had earlier publication dates than the other SRs included, suggesting that there may have been variations in the requirements and standards for conducting SRs at that time. Additionally, this observation implies that over time, there is a trend towards conducting more robust and higher‐quality qualitative SRs in this field.

In evaluating the association between PerioD and CVD/CVE, this synthesis applies both the Bradford Hill criteria [18] and the GRADE [35] approach, revealing an overlap between the two frameworks (Tables 5 and 6). A previous publication highlights this finding, indicating the ongoing influence of the Bradford Hill criteria [18] and the use of other approaches, such as GRADE [35] for understanding the viewpoints of Bradford Hill [18]. By integrating GRADE [35] principles, it becomes evident why the criterion of specificity may have limited applicability in causal inference. When it comes to causal assessment the need for an enhanced clarity and standardisation in the field of epidemiology has previously been noted [82]. The Bradford Hill criteria [18] continue to hold a fundamental importance in causal assessment and SRs and epidemiological studies consistently demonstrate an association between PerioD and CVD/CVE [82]. This synthesis assessed the causality of the relationship between PerioD and CVD/CVE, applying the Bradford Hill criteria [18]. The consistent findings across SRs, the presence of a biological gradient and the plausibility of shared mechanisms all may suggest a potential causal link. However, the magnitude of the association could be considered mostly negligible to small. Additionally, other criteria such as specificity, temporality, experimental evidence and analogy were not fully explored in this synthesis, limiting the assessment of causality. It is important to acknowledge that the present findings do not provide definitive evidence for establishing causality. The findings from the assessment of Bradford Hill criteria [18] support the analysis in a recent narrative review that critically appraised the association between PerioD and ACVD [67]. The authors of this review concluded that, based on Bradford Hill's criteria [18], no definitive conclusion can be drawn regarding the causal relationship between PerioD and ACVD [67]. As of now, there is a lack of evidence addressing the crucial issue of the temporal relationship between exposures and outcomes.

It remains to be determined whether future investigations will yield a positive estimate for meeting Bradford Hill criteria [18]. Establishing causality through intervention studies remains challenging. The lack of well‐designed, blinded randomised controlled trials with CVD/CVE outcomes is a significant limitation. Conducting high‐quality, double‐blinded, randomised controlled trials with PerioD and CVD/CVE outcomes is on the other hand complicated due to ethical considerations.

Furthermore, the discussion surrounding the association between PerioD and CVD raises intriguing points. According to SR VI [44], the absence of a clear causal relationship between PerioD and CHD suggests the possibility of PerioD serving as a risk marker rather than a direct cause. This perspective introduces the concept of unexplained confounding, where a factor connected to both PerioD and CHD, such as smoking, diet, DM, or socio‐economic status, may contribute to the observed association. SR VIII [46] reaffirms this viewpoint, suggesting that reported positive associations could be influenced by biases or residual confounding, given shared risk factors like age, smoking, stress, socio‐economic status, body fat content and health consciousness. Consequently, the review authors of SR VIII [46] express uncertainty about whether the association between PerioD and CVD is specific or coincidental. Furthermore, SR XVI [54] acknowledges an association between PerioD and CVD but emphasises that the causal mechanism underlying this link is not yet well‐established. These discussions collectively underscore the complexity of the relationship between PerioD and CVD, urging further research to clarify the intricacies and nature of their association.

### 4.8 Limitations

- In this synthesis, the decision to exclusively include individuals aged ≥ 16 years, excluding those < 16 years, was made due to the acknowledged significant role of genetics in the development of both PerioD and CVD/CVE, particularly at a younger age [72]. This focus aimed to mitigate the potential confounding effects of genetic factors on the observed association between PerioD and CVD/CVE parameters.
- In this synthesis, CVD/CVE were defined as CVD or CVE, with a focus on specific and clinically relevant evidence for the potential association. However, excluding parameters like HT, AF, arterial stiffness, arterial calcification and surrogate markers for CVD/CVE outcomes may limit the comprehensive understanding of the relationship between PerioD and CVD/CVE. While these parameters are established risk factors for CVD/CVE, their exclusion could overlook important subclinical manifestations or early indicators of cardiovascular damage relevant to PerioD [11, 83, 84, 85, 86, 87, 88, 89]. Conversely, by excluding these intermediate parameters and relying on actual CVD and CVE, the synthesis ensures a more robust assessment of the direct impact, providing specific and clinically relevant evidence on the association between PerioD and CVD/CVE.
- It is also important to recognise that both PerioD and CVD/CVE are multifactorial conditions, with their development involving a complex interplay of various causal components [14, 90].
- DM, a metabolic disease, adversely affects periodontal and cardiovascular health through microvascular changes and endothelial dysfunction [14, 90]. Complications impact bone health and contribute significantly to PerioD. DM is a confirmed risk factor for PerioD and CVD/CVE [14, 90, 91, 92, 93]. Excluding DM patients in this synthesis clarifies the direct association but limits broader applicability. By omitting this population, the review may fall short of fully capturing the potential impact of PerioD on cardiovascular health in real‐world settings, where patients having DM and metabolic syndrome are common.
- As this synthesis of SRs analysed pooled ORs and RRs from included SRs, direct comparisons of meta‐analyses outcomes between studies may yield inconsistent interpretations of association strength [94]. The interchangeability of fixed and random effects models in these studies poses a challenge for direct comparisons, as these models make different assumptions about underlying variability [94, 95]. Therefore, when assessing SRs using different models and outcome parameters, the potential impact of these methodological differences on the findings should be considered.
- A limitation of the synthesis of SRs lies in the use of meta‐analysis for observational studies. While a meta‐analysis proves valuable for comprehending variability across studies, it introduces challenges due to inherent biases and divergent study designs. The potential for publication bias further complicates the synthesis, emphasising the need for cautious interpretation and consideration of these limitations in drawing conclusions from the merged findings.

### 4.9 Directions of Future Research

For future investigations into the association between PerioD and CVD/CVE, there is a need for standardised definitions of both conditions and meticulous adjustment for confounding factors. This approach is essential to gain a more comprehensive understanding of their relationship. Moreover, beyond these considerations, a priority should be placed on exploring the biological mechanisms. This emphasis aims to enhance our understanding of the potential causal relationship between PerioD and CVD/CVE.

## 5 Conclusion

This synthesis of SRs provides insights into the association between PerioD and CVD/CVE. With moderate certainty, the magnitude of this association can generally be estimated as negligible to small, and causality, according to Bradford Hill criteria, remains undefined. It is noteworthy that factors such as gender, age, PerioD severity, smoking status and geographic region may be confounders in the association between PerioD and CVD/CVE. This nuanced understanding underscores the complexity of the relationship and emphasises the need for ongoing exploration in future research.

## 6 Clinical Relevance

### 6.1 Scientific Rationale for This Analysis

Given the high prevalence of PerioD and CVD/CVE and their shared risk factors, there is a growing interest in understanding the association between these conditions.

### 6.2 Principle Findings

Regarding the relationship between PerioD and CVD/CVE, there is moderate certainty indicating a negligible to small association. Therefore, the link between PerioD and CVD/CVE remains inconclusive, and causality in their relationship has not yet been definitively identified.

### 6.3 Practical Implications

In the absence of a potential association, it remains important to maintain good oral health for overall well‐being, as PerioD can also negatively affect the quality of life.

## Author Contributions

**Max G.P. Schoenmakers:** contributed to design, search and selection, analysis and interpretation and drafted the manuscript. **Lotte P.M. Weijdijk:** contributed to conception and design, analysis and interpretation and critically revised the manuscript. **Eveline J.S. Willems:** contributed to search and selection and critically revised the manuscript. **Fridus (G.A.) van der Weijden:** contributed to conception and design, analysis and interpretation and critically revised the manuscript. **Dagmar Else Slot:** contributed to conception and design, search and selection, analysis and interpretation and critically revised the manuscript. All authors gave final approval and agreed to be accountable for all aspects of work ensuring integrity and accuracy.

## Ethics Statement

This research has been approved by the ACTA Institutional review board, by reference number 2022–74229 and registered at the International Prospective Register of Systematic Reviews (PROSPERO) by number CRD42023444999.

## Conflicts of Interest

The authors declare no conflicts of interest.

## Supporting information


**Appendix S1.** Excluded studies based on selection criteria (*N* = 11).
**Appendix S2**. Estimated the risk of bias by scoring a list of items related to the reporting and methodological quality of the included SRs.
**Appendix S3**. Citation matrix.
**Appendix S4**. Detailed analysis of the different outcome aspects.
**Appendix S5**. Detailed analysis of the Bradford Hill criteria [18].
**Appendix S6**. List of abbreviations.
**Appendix S7**. JBI [19] checklist.
**Appendix S8**. MOOSE [20] checklist.

## Data Availability

The data that supports the findings of this study are available in the Supporting Information of this article.
