# Supplementary material for: The Association of Periodontitis With Cardiovascular Disease Parameters: A Synthesis of Systematic Reviews
Source: Int J Dent Hyg. 2026 Feb 24;24(2):278–306. doi: 10.1111/idh.12885 (PMC13050389; doi:10.1111/idh.12885)
Supplement: Supplementary file 1 — Appendix S1. Excluded studies based on selection criteria (N = 11). Appendix S2. Estimated the risk of bias by scoring a list of items related to the reporting and methodological quality of the included SRs. Appendix S3. Citation matrix. Appendix S4. Detailed analysis of the different outcome aspects. Appendix S5. Detailed analysis of the Bradford Hill criteria [18]. Appendix S6. List of abbreviations. Appendix S7. JBI [19] checklist. Appendix S8. MOOSE [20] checklist. [file IDH-24-278-s001.docx]

To be published in: The International Journal of Dental Hygiene

Version: 2024-01-04

**Onlina Appendix from:**

**The association of periodontitis with cardiovascular disease parameters - a synthesis of systematic reviews-**

Max G.P. Schoenmakers^1^ (https://orcid.org/0000-0002-1783-1439)

Lotte P.M. Weijdijk^1,2^ (https://orcid.org/0000-0001-7910-9778)

Eveline J.S. Willems^1^ (https://orcid.org/0000-0002-4429-6048)

Fridus (G.A.) van der Weijden^1^ (https://orcid.org/0000-0002-5075-8384)

Dagmar Else Slot^1^ (https://orcid.org/0000-0001-7234-0037)

**Author affiliations:**

^1^ Department of Periodontology Academic Center for Dentistry Amsterdam (ACTA), ACTA is a joint venture between the Faculty of Dentistry of the University of Amsterdam

^2^ Department of Oral and Maxillofacial Surgery, Amsterdam UMC and Academic Centre for Dentistry Amsterdam (ACTA), ACTA is a joint venture between the Faculty of Dentistry of the University of Amsterdam and the Faculty of Dentistry of the Vrije Universiteit Amsterdam, Amsterdam, The Netherlands.

CONFLICT OF INTEREST AND SOURCE OF FUNDING STATEMENT:

The first author and co-authors declare that they have no conflicts of interest.

This paper was prepared as part of the obligation of the first author to fulfil the requirements of the ACTA Master’s program in dentistry. This review was self-funded by the authors and their institution.

This research has been approved by the ACTA Institutional review board,

by reference number 2022-74229 and registered at the International Prospective Register of Systematic Reviews (PROSPERO) by number CRD42023444999.

**Online Appendix S1**

Excluded studies based on selection criteria (N=11).

Excluded studies (N=11) after the full text assessment of the search and selection procedure. These studies were assessed based on the defined eligibility criteria.

| N/A study design | 1. Matthews 2008^1^ 2. Froum et al. 2023^2^ 3. Cronin 2009^3^ 4. Leng et al. 2015^4^ 5. Meurman et al. 2004^5^ 6. Choi et al. 2021^6^ 7. Baniulyte et al. 2021^7^ |
| --- | --- |
| Wrong population | 1. Dewan et al. 2023^8^ 2. Leng et al. 2023^9^ 3. Guo et al. 2023^10^ 4. Alwithanani 2023^11^ |

**References:**

1. Matthews D. Possible link between periodontal disease and coronary heart disease. Evid Based Dent [Internet]. 2008 [cited 2023 Mar 30];(9):8. Available from: https://doi.org/ 10.1038/sj.ebd.6400560
2. Froum SJ, Hengjeerajaras P, Liu KY, Maketone P, Patel V, Shi Y. The Link Between Periodontitis/Peri-implantitis and Cardiovascular Disease: A Systematic Literature Review. Int J Periodontics Restorative Dent [Internet]. 2020;40(6):e229–33. Available from: https://pubmed.ncbi.nlm.nih.gov/33151189/
3. Cronin A. Periodontal disease is a risk marker for coronary heart disease? Evidence-Based Dentistry [Internet]. 2009 [cited 2023 Mar 30];(10):22. Available from: https://doi.org/ 10.1038/sj.ebd.6400634
4. Leng WD, Zeng XT, Kwong JSW, Hua XP. Periodontal disease and risk of coronary heart disease: An updated meta-analysis of prospective cohort studies. Int J Cardiol. 2015 Oct 10;201:469–72.
5. Meurman JH, Sanz M, Janket SJ. Oral health, atherosclerosis, and cardiovascular disease. Crit Rev Oral Biol Med [Internet]. 2004;15(6):403–13. Available from: https://pubmed.ncbi.nlm.nih.gov/15574681/
6. Choi H, Dey AK, Priyamvara A, Aksentijevich M, Bandyopadhyay D, Dey D, et al. Role of Periodontal Infection, Inflammation and Immunity in Atherosclerosis. Curr Probl Cardiol [Internet]. 2021;46(3):100638. Available from: https://pubmed.ncbi.nlm.nih.gov/32646544/
7. Baniulyte G, Piela K, Culshaw S. How strong is the link between periodontitis and stroke? Evidence-Based Dentistry [Internet]. 2021 [cited 2023 Mar 30];(22):10–1. Available from: https://doi.org/ 10.1038/s41432-021-0161-7
8. Dewan M, Pandit A, Goyal L. Association of periodontitis and gingivitis with stroke: A systematic review and meta-analysis. Dent Med Probl. 2023 Jan 26;61(3):0–0.
9. Leng Y, Hu Q, Ling Q, Yao X, Liu M, Chen J, et al. Periodontal disease is associated with the risk of cardiovascular disease independent of sex: A meta-analysis. Front Cardiovasc Med. 2023 Feb 27;10.
10. Guo X, Li X, Liao C, Feng X, He T. Periodontal disease and subsequent risk of cardiovascular outcome and all-cause mortality: A meta-analysis of prospective studies. *PLoS One*. 2023;18(9):e0290545. doi:10.1371/JOURNAL.PONE.0290545
11. Alwithanani N. Periodontal Diseases and Heart Diseases: A Systemic Review. *J Pharm Bioallied Sci*. 2023;15(Suppl 1):S72. doi:10.4103/JPBS.JPBS_517_22

**Online Appendix S2**

Estimated the risk of bias by scoring a list of items related to the reporting and methodological quality of the included SRs.

| Author  Criteria | I: Bahekar et al. 2007 ^12^ | II: Blaizot et al. 2009 ^13^ | III: Dietrich et al. 2013 ^14^ | IV: Fagundes et al. 2019 ^15^ | V: Gao et al. 2021 ^16^ | VI: Humphrey et al. 2008 ^17^ | VII: Janket et al. 2003 ^18^ | VIII: Khader et al. 2004 ^19^ | IX: Lafon et al. 2014 ^20^ | X: Larvin et al. 2020 ^21^ | XI: Leira et al. 2017 ^22^ | XII: Meregildo-Rodriguez et al. 2022 ^23^ | XIII: Orlandi et al. 2014 (15) | XIV: Qin et al. 2021 (16) | XV: Sfyroeras et al. 2012 (18) | XVI: Voinescu et al. 2019 ^26^ | XVII: Wang et al. 2019 (20) | XVIII: Xu et al. 2017 (22) | XIX: Zeng et al. 2015 (24) |
| --- | --- | --- | --- | --- | --- | --- | --- | --- | --- | --- | --- | --- | --- | --- | --- | --- | --- | --- | --- |
| Defined outcome criteria of interest | + | + | + | + | + | + | + | + | + | + | + | + | + | + | + | + | + | + | + |
| Describes the rationale | + | + | + | + | + | + | + | + | + | + | + | + | + | + | + | + | + | + | + |
| Describes the focused (PICO)[S] question/ hypothesis | + | + | + | + | + | + | + | + | + | + | + | + | + | + | + | + | + | + | + |
| Describes if a protocol was developed ‘a pirori’. | - | + | - | + | + | - | - | - | - | + | + | + | + | - | - | + | + | + | - |
| Protocol registration/publication | - | - | - | + | + | - | - | - | - | + | + | + | - | - | - | - | - | - | - |
| Presented eligibility criteria (in/exclusion criteria) | + | + | + | + | + | + | + | + | + | + | + | + | + | + | + | + | + | + | + |
| Presents the full search strategy | - | + | + | + | + | - | - | - | + | + | + | + | + | + | - | - | - | + | + |
| Various databases searched | + | + | - | + | + | - | - | - | + | + | + | + | + | + | - | + | + | + | + |
| Performed (hand) search in additional sources (f.i. grey literature or trial registers) | + | + | - | + | - | - | - | + | + | + | + | + | + | - | + | + | + | + | - |
| Review selection by more than 1 reviewer | + | + | +- | + | + | + | - | + | + | + | + | + | + | + | + | + | + | + | + |
| Non-English papers included | - | + | + | + | - | - | - | - | + | - | - | + | + | - | - | + | - | - | - |
| Provide details on the performed study selection process/ flow chart | + | + | + | + | + | + | + | + | + | + | + | + | + | + | + | + | + | + | + |
| Report included study characteristics | + | + | + | + | + | + | + | + | + | + | + | + | + | + | + | + | + | + | + |
| Provide data of the selected studies on the outcome measures of interest | + | + | + | + | + | + | + | + | + | + | + | + | + | + | + | + | + | + | + |
| Data were extracted by more than 1 reviewer | + | + | + | + | + | + | + | + | - | + | + | - | + | + | + | + | - | + | + |
| Contacted authors for additional information | - | + | - | + | - | - | - | - | - | - | + | - | + | - | - | - | - | - | - |
| Report heterogeneity of the included studies | + | + | + | + | + | + | + | + | + | + | + | + | + | + | + | + | + | + | + |
| Estimated risk of bias in individual studies | + | + | - | + | + | + | + | - | + | + | + | + | + | + | - | - | + | + | - |
| Performed a meta analysis | + | + | - | + | + | + | + | + | + | + | + | + | + | + | + | - | + | + | + |
| Performed a descriptive analysis | - | - | + | - | - | + | - | - | - | - | + | - | + | - | - | + | - | - | - |
| Describe additional sub analysis | + | + | + | + | + | + | + | + | + | + | + | + | + | + | - | + | + | + | + |
| Grading of the obtained evidence | - | - | - | + | - | - | - | - | - | - | - | - | - | - | - | - | - | - | - |
| Present limitations of the systematic review | + | + | + | + | + | + | - | + | + | + | + | + | +/- | + | + | - | + | + | + |
| Provide a conclusion that respond to the objective | + | + | + | + | + | + | + | + | + | + | + | + | + | + | + | + | + | + | + |
| Publication bias assessed | - | + | - | + | + | + | + | + | - | + | + | + | + | + | + | - | + | + | + |
| Funding source | - | - | + | + | + | + | - | - | - | + | + | + | + | + | + | + | + | + | + |
| Conflict of interest statement | - | - | + | + | + | + | - | - | + | + | + | + | + | + | + | + | + | + | + |
| **Original authors estimated level of evidence** | **NR** | **NR** | **NR** | **Low** | **NR** | **NR** | **NR** | **NR** | **NR** | **NR** | **NR** | **NR** | **NR** | **NR** | **NR** | **NR** | **NR** | **NR** | **NR** |
| Current authors estimated quality score ^30^ | 63% | 81% | 69% | 96% | 81% | 70% | 52% | 59% | 70% | 85% | 89% | 85% | 91% | 74% | 63% | 63% | 74% | 81% | 70% |
| **Current authors estimated risk of bias** | **Moderate** | **Low** | **Moderate** | **Low** | **Low** | **Moderate** | **Substantial** | **Substantial** | **Moderate** | **Low** | **Low** | **Low** | **Low** | **Moderate** | **Moderate** | **Moderate** | **Moderate** | **Low** | **Moderate** |

Abbreviations: NR, not reported.

Each aspect of the reporting and methodological quality item score list was given a rating of a plus ‘+’ for informative description of the item at issue and a study design meeting the quality standard, was assigned, plus-minus (±) was assigned if the item was incompletely described and minus ‘-’ was used if the item was not described or unknown ^31^. For the quality assessment score individual items with a positive rating were summed to obtain an overall percentage score ^30^.

**Online Appendix S3**

Citation matrix, an overview of included SRs and their corresponding primary studies.

| Included  SRs  Included  studies | Janket et al. 2003 (VII) | Khader et al. 2004 (VIII) | Bahekar et al. 2007 (I) | Humphrey et al. 2008 ((VI) | Blaizot et al. 2009 (II) | Sfyroeras et al. 2012 (XV) | Dietrich et al. 2013 (III) | Lafon et al. 2014 (IX) | Orlandi et al. 2014 (XIII) | Zeng et al. 2015 (XIX) | Leira et al. 2017 (XI) | Xu et al. 2017 (XVIII) | Wang et al. 2019 (XVII) | Voinescu et al. 2019 (XVI) | Fagundes et al. 2019 (IV) | Larvin et al. 2020 (X) | Gao et al. 2021 (V) | Qin et al. 2021 (XIV) | Meregildo-Rodriguez et al. 2022 (XII) |
| --- | --- | --- | --- | --- | --- | --- | --- | --- | --- | --- | --- | --- | --- | --- | --- | --- | --- | --- | --- |
| DeStefano et al. 1993 | x | x | x | x | x |  |  |  |  |  |  |  |  |  |  | x | x |  | x |
| Mattila et al. 1995 | x |  | x |  |  |  |  |  |  |  |  |  |  |  |  |  |  |  |  |
| Beck et al. 1996 | x | x |  |  |  | x |  |  |  |  | x |  |  |  |  | x |  |  | x |
| Joshipura et al. 1996 | x | x | x | x |  |  |  |  |  |  |  | x |  |  |  | x | x | x | x |
| Genco et al. 1997 | x |  |  |  |  |  |  |  |  |  |  |  |  |  |  |  |  |  |  |
| Grau et al. 1997 |  | x |  |  |  |  |  |  |  |  |  |  |  |  |  |  |  |  |  |
| Loesche et al. 1998 |  | x | x |  | x |  |  |  |  |  |  |  |  |  |  |  |  |  |  |
| Mendez et al. 1998 |  |  |  |  |  |  | x |  |  |  |  |  | x |  |  |  |  |  |  |
| Arbes et al. 1999 |  | x | x |  | x |  |  |  |  |  |  |  |  |  |  |  |  |  | x |
| Morrison et al. 1999 | x | x |  | x | x |  |  | x |  |  |  |  |  |  |  | x | x |  | x |
| Wu et al. 1999 | x |  | x |  |  |  |  |  |  |  |  |  |  |  |  |  |  |  | x |
| Hujoel et al. 2000 | x | x |  |  |  |  |  |  |  |  |  |  |  |  |  |  | x |  | x |
| Wu et al. 2000 |  | x |  |  |  | x |  | x |  |  | x |  |  |  | x | x | x |  |  |
| Beck et al. 2001 |  |  |  |  |  |  |  |  | x | x |  |  | x |  |  |  |  |  |  |
| Howell et al. 2001 | x | x | x | x |  | x |  | x |  |  |  | x |  |  |  | x | x | x | x |
| Hujoel et al. 2001 |  |  |  |  |  |  |  |  |  |  |  |  |  |  |  | x |  |  |  |
| Katz et al. 2001 |  |  |  |  | x |  |  |  |  |  |  |  |  |  |  |  |  |  |  |
| Bazile et al. 2002 |  |  |  |  |  |  |  |  |  |  |  | x |  |  |  |  |  |  | x |
| Bloemenkamp et al. 2002 |  |  |  |  |  |  |  |  |  |  |  |  | x |  |  |  |  |  |  |
| Buhlin et al. 2002 |  | x | x |  | x |  |  |  |  |  |  | x |  |  |  |  |  |  |  |
| Jansson et al. 2002 |  |  |  |  | x |  |  |  |  |  |  |  |  |  |  |  |  |  |  |
| Lopez et al. 2002 |  |  |  |  |  |  | x |  |  |  |  |  |  |  |  |  |  |  |  |
| Malthaner et al. 2002 |  |  | x |  | x |  |  |  |  |  |  |  |  |  |  |  |  |  |  |
| Persson et al. 2002 |  |  |  |  |  |  |  |  |  | x |  |  | x |  |  |  |  |  |  |
| Ajwani et al. 2003 |  |  |  | x |  |  | x |  |  |  |  |  |  |  |  |  |  |  |  |
| Amar et al. 2003 |  |  |  |  |  |  |  |  | x |  |  |  |  |  |  |  |  |  |  |
| Buhlin et al. 2003 |  |  | x |  | x |  |  |  |  |  |  |  |  |  |  |  |  |  |  |
| Elter et al. 2003 |  |  |  |  |  | x |  |  |  |  |  |  |  |  |  |  |  |  |  |
| Frisk et al. 2003 |  |  |  |  | x |  |  |  |  |  |  |  |  |  |  |  |  |  |  |
| Hung et al. 2003 |  |  |  |  |  |  |  |  |  |  |  |  | x |  |  | x |  |  |  |
| Joshipura et al. 2003 |  |  |  |  |  | x |  | x |  |  |  |  |  |  |  | x |  |  |  |
| Nicolosi et al. 2003 |  |  |  |  | x |  |  |  |  |  |  |  |  |  |  |  |  |  |  |
| Persson et al. 2003 |  |  |  |  |  |  |  |  |  |  |  | x |  |  |  |  |  |  |  |
| Ravon et al. 2003 |  |  |  |  |  |  |  |  |  | x |  |  | x |  |  |  |  |  |  |
| Rutger et al. 2003 |  |  |  |  |  |  |  |  |  |  |  |  |  |  |  |  |  |  | x |
| Tuominen et al. 2003 |  |  |  | x | x |  | x |  |  |  |  |  |  |  |  |  |  |  |  |
| Desvarieux et al. 2004 |  |  |  |  |  |  |  |  | x | x |  |  |  |  |  |  |  |  |  |
| Dörfer et al. 2004 |  |  |  |  |  | x |  |  |  |  | x |  |  |  | x |  |  |  |  |
| Elter et al. 2004 |  |  | x |  | x |  |  |  |  |  |  |  |  |  |  |  |  |  |  |
| Geerts et al. 2004 |  |  | x |  | x |  |  |  |  |  |  |  |  |  |  |  |  |  | x |
| Grau et al. 2004 |  |  |  |  |  | x | x |  |  |  | x |  |  |  |  |  |  |  |  |
| Hung et al. 2004 |  |  |  | x | x |  |  |  |  |  |  |  |  |  |  | x | x |  |  |
| Mercanoglu et al. 2004 |  |  |  |  |  |  |  |  | x |  |  |  |  |  |  |  |  |  |  |
| Montebugnoli et al. 2004 |  |  |  |  | x |  |  |  |  |  |  |  |  |  |  |  |  |  | x |
| Renvert et al. 2004 |  |  |  |  | x |  |  |  |  |  |  |  |  |  |  |  |  |  |  |
| Abnet et al. 2005 |  |  |  |  | x | x |  | x |  |  |  |  |  |  |  | x |  |  |  |
| Beck et al. 2005 |  |  |  |  |  |  |  |  | x |  |  |  |  |  |  |  |  |  |  |
| Buhlin et al. 2005 |  |  | x |  | x |  |  |  |  |  |  |  |  |  |  |  |  |  | x |
| Cabrera et al. 2005 |  |  |  |  | x |  |  |  |  |  |  |  |  |  |  |  |  |  |  |
| Coelho et al. 2005 |  |  |  |  | x |  |  |  |  |  |  |  |  |  |  |  |  |  | x |
| Cueto et al. 2005 |  |  |  |  | x |  | x |  |  |  |  | x |  |  |  |  |  |  | x |
| Desvarieux et al. 2005 |  |  |  |  |  |  |  |  | x |  |  |  | x |  |  |  |  |  |  |
| Engebretson et al. 2005 |  |  |  |  |  |  |  |  |  | x |  |  | x |  |  |  |  |  |  |
| Leivadarios et al. 2005 |  |  |  |  |  |  |  |  | x |  |  |  | x |  |  |  |  |  |  |
| Pussinen et al. 2005 |  |  |  |  |  |  |  |  | x |  |  |  |  |  |  |  |  |  |  |
| Seinost et al. 2005 |  |  |  |  |  |  |  |  | x |  |  |  |  |  |  |  |  |  |  |
| Söder et al. 2005 |  |  |  |  |  |  |  |  | x | x |  |  | x |  |  |  |  |  |  |
| Andriankaja et al. 2006 |  |  |  |  | x |  |  |  |  |  |  | x |  |  |  |  |  |  | x |
| Barilli et al. 2006 |  |  |  |  | x |  |  |  |  |  |  |  |  |  |  |  |  |  |  |
| Briggs et al. 2006 |  |  | x |  | x |  |  |  |  |  |  |  |  |  |  |  |  |  | x |
| Elter et al. 2006 |  |  |  |  |  |  |  |  | x |  |  |  |  |  |  |  |  |  |  |
| Franek et al. 2006 |  |  |  |  |  |  |  |  | x |  |  |  |  |  |  |  |  |  |  |
| Geismar et al. 2006 |  |  |  |  | x |  |  |  |  |  |  |  |  |  |  |  |  |  | x |
| Holmlund et al. 2006 |  |  |  |  | x |  |  |  |  |  |  | x |  |  |  |  |  |  | x |
| Lee et al. 2006 |  |  |  |  |  | x |  |  |  |  |  |  |  |  |  |  |  |  |  |
| Rech et al. 2006 |  |  |  |  | x |  |  |  |  |  |  |  |  |  |  |  |  |  |  |
| Schillinger et al. 2006 |  |  |  |  |  |  |  |  |  | x |  |  |  |  |  |  |  |  |  |
| Spahr et al. 2006 |  |  | x |  |  |  |  |  |  |  |  |  |  |  |  |  |  |  | x |
| Andriankaja et al. 2007 |  |  |  |  |  |  | x |  |  |  |  | x |  |  |  |  |  |  | x |
| Blum et al. 2007 |  |  |  |  |  |  |  |  | x |  |  |  |  |  |  |  |  |  |  |
| Gotsman et al. 2007 |  |  |  |  | x |  |  |  |  |  |  |  |  |  |  |  |  |  |  |
| Latronico et al. 2007 |  |  |  |  | x |  |  |  |  |  |  |  |  |  |  |  |  |  |  |
| Nonnenmacher et al. 2007 |  |  |  |  | x |  |  |  |  |  |  |  |  |  |  |  |  |  | x |
| Recht et al. 2007 |  |  |  |  |  |  |  |  |  |  |  |  |  |  |  |  |  |  | x |
| Söder et al. 2007 |  |  |  |  |  |  |  |  | x |  |  |  |  |  |  |  |  |  |  |
| Tonetti et al. 2007 |  |  |  |  |  |  |  |  | x |  |  |  |  | x |  |  |  |  |  |
| Tu et al. 2007 |  |  |  |  |  |  |  | x |  |  |  |  |  |  |  | x |  |  |  |
| Cairo et al. 2008 |  |  |  |  |  |  |  |  | x |  |  |  |  |  |  |  |  |  |  |
| Chen et al. 2008 |  |  |  |  |  |  |  |  |  |  |  |  | x |  |  |  |  |  |  |
| Demmer et al. 2008 |  |  |  |  |  |  |  |  | x |  |  |  |  |  |  |  |  |  |  |
| Dietrich et al. 2008 |  |  |  |  |  |  | x |  |  |  |  |  |  |  |  | x |  |  |  |
| Heitmann et al. 2008 |  |  |  |  |  |  |  | x |  |  |  |  |  |  |  | x |  |  |  |
| Higashi et al. 2008 |  |  |  |  |  |  |  |  | x |  |  |  |  |  |  |  |  |  |  |
| Senba et al. 2008 |  |  |  |  |  |  |  |  |  |  |  | x |  |  |  |  |  |  |  |
| Sim et al. 2008 |  |  |  |  |  | x | x |  |  |  | x |  |  |  |  |  |  |  |  |
| Starkmammart et al. 2008 |  |  |  |  |  |  |  |  |  |  |  |  |  |  |  |  |  |  | x |
| Cairo et al. 2009 |  |  |  |  |  |  |  |  | x |  |  |  |  |  |  |  |  |  |  |
| Choe et al. 2009 |  |  |  |  |  |  |  |  |  |  |  |  |  |  |  | x |  |  |  |
| Higashi et al. 2009 |  |  |  |  |  |  |  |  | x |  |  |  |  |  |  |  |  |  |  |
| Jimenez et al. 2009 |  |  |  |  |  | x | x | x |  |  | x |  |  |  |  | x |  |  |  |
| Li et al. 2009 |  |  |  |  |  |  |  |  | x |  |  |  |  |  |  |  |  |  |  |
| Moher et al. 2009 |  |  |  |  |  |  |  |  |  |  |  |  |  | x |  |  |  |  |  |
| Mucci et al. 2009 |  |  |  |  |  |  |  |  |  |  |  |  |  |  |  | x | x |  |  |
| Oe et al. 2009 |  |  |  |  |  |  |  |  |  |  |  |  |  |  |  |  |  |  | x |
| Piconi et al. 2009 |  |  |  |  |  |  |  |  | x |  |  |  |  |  |  |  |  |  |  |
| Söder et al. 2009 |  |  |  |  |  |  |  |  | x |  |  |  |  |  |  |  |  |  |  |
| Carallo et al. 2010 |  |  |  |  |  |  |  |  |  | x |  |  | x |  |  |  |  |  |  |
| Coelho et al. 2010 |  |  |  |  |  |  |  |  |  |  |  |  |  |  |  |  |  |  | x |
| Dorn et al. 2010 |  |  |  |  |  |  | x |  |  |  | x |  |  |  |  |  |  | x | x |
| Holmlund et al. 2010 |  |  |  |  |  |  |  | x |  |  |  |  |  |  | x |  | x |  |  |
| Kim et al. 2010 |  |  |  |  |  | x |  |  |  |  |  |  |  |  |  |  |  |  |  |
| Pradeep et al. 2010 |  |  |  |  |  | x |  |  |  |  | x |  |  |  | x |  |  |  |  |
| Renvert et al. 2010 |  |  |  |  |  |  |  |  |  |  |  | x |  |  |  |  |  |  |  |
| Ylostalo et al. 2010 |  |  |  |  |  |  |  |  | x |  |  |  |  |  |  |  |  |  |  |
| Abolfazli et al. 2011 |  |  |  |  |  |  |  |  |  |  |  |  |  |  | x |  |  |  |  |
| Anyaipoma et al. 2011 |  |  |  |  |  |  |  |  |  |  |  |  |  |  |  |  |  |  | x |
| Holmlund et al. 2011 |  |  |  |  |  |  |  |  |  |  |  | x |  |  |  |  |  |  | x |
| Li et al. 2011 |  |  |  |  |  |  |  |  | x |  |  |  |  |  |  |  |  |  |  |
| Lopez et al. 2011 |  |  |  |  |  |  |  |  | x |  |  |  |  |  |  |  |  |  |  |
| Viera et al. 2011 |  |  |  |  |  |  |  |  | x |  |  |  |  |  |  |  |  |  |  |
| Xu & Lu 2011 |  |  |  |  |  |  | x |  |  |  |  |  |  |  |  |  |  |  |  |
| Franek et al. 2012 |  |  |  |  |  |  |  |  | x |  |  |  |  |  |  |  |  |  |  |
| Ghizoni et al. 2012 |  |  |  |  |  |  |  |  |  |  |  |  |  |  | x |  |  |  |  |
| Lopez-Jornet et al. 2012 |  |  |  |  |  |  |  |  | x | x |  |  | x |  |  |  |  |  |  |
| Puhar et al. 2012 |  |  |  |  |  |  |  |  | x |  |  |  |  |  |  |  |  |  |  |
| Rivas-Tumanyan et al. 2012 |  |  |  |  |  |  |  |  |  |  |  |  |  |  |  | x |  |  |  |
| Southerland et al. 2012 |  |  |  |  |  |  |  |  | x |  |  |  |  |  |  |  |  |  |  |
| Zahnd et al. 2012 |  |  |  |  |  |  |  |  | x |  |  |  |  |  |  |  |  |  |  |
| Hayashida et al. 2013 |  |  |  |  |  |  |  |  | x | x |  |  | x |  |  |  |  |  |  |
| Khosravi et al. 2013 |  |  |  |  |  |  |  |  |  |  |  | x |  |  |  |  |  |  | x |
| Lee et al. 2013 |  |  |  |  |  |  |  |  |  |  |  |  |  |  |  | x |  |  |  |
| Li et al. 2013 |  |  |  |  |  |  |  |  |  |  |  | x |  |  |  |  |  |  | x |
| Parkar et al. 2013 |  |  |  |  |  |  |  |  |  |  |  |  |  |  |  |  |  |  | x |
| Pinho et al. 2013 |  |  |  |  |  |  |  |  | x | x |  |  | x |  |  |  |  |  |  |
| Ramesh et al. 2013 |  |  |  |  |  |  |  |  |  | x |  |  | x |  |  |  |  |  |  |
| Ruiz et al. 2013 |  |  |  |  |  |  |  |  | x |  |  |  |  |  |  |  |  |  |  |
| Sotto-Barreras et al. 2013 |  |  |  |  |  |  |  |  |  |  |  |  | x |  |  |  |  |  |  |
| Sujal et al. 2013 |  |  |  |  |  |  |  |  |  |  |  | x |  |  |  |  |  |  |  |
| Vidal et al. 2013 |  |  |  |  |  |  |  |  |  |  |  |  |  |  |  |  |  |  |  |
| Wozakowska-Kaplon et al. 2013 |  |  |  |  |  |  |  |  |  | x |  |  | x |  |  |  |  |  |  |
| de Boer et al. 2014 |  |  |  |  |  |  |  |  |  |  |  |  |  | x |  |  |  |  |  |
| Carallo et al. 2014 |  |  |  |  |  |  |  |  |  |  |  |  |  | x |  |  |  |  |  |
| Heaton et al. 2014 |  |  |  |  |  |  |  |  |  |  |  |  |  | x |  |  |  |  |  |
| Jeffcoat et al. 2014 |  |  |  |  |  |  |  |  |  |  |  |  |  | x |  |  |  |  |  |
| Jung et al. 2014 |  |  |  |  |  |  |  |  |  | x |  |  | x |  |  |  |  |  |  |
| Kodovazenitis et al. 2014 |  |  |  |  |  |  |  |  |  |  |  | x |  |  |  |  |  |  |  |
| Lafon et al. 2014 |  |  |  |  |  |  |  |  |  |  | x |  |  |  |  |  |  |  |  |
| Noguchi et al. 2014 |  |  |  |  |  |  |  |  |  |  |  |  |  |  |  | x | x | x | x |
| Palm et al. 2014 |  |  |  |  |  |  |  |  |  |  |  |  |  |  | x |  |  |  |  |
| WIllershausen et al. 2014 |  |  |  |  |  |  |  |  |  |  |  | x |  |  |  |  |  |  | x |
| Yu et al. 2014 |  |  |  |  |  |  |  |  |  | x |  |  | x |  |  |  |  |  |  |
| Choe et al. 2015 |  |  |  |  |  |  |  |  |  |  |  |  |  |  |  | x |  |  |  |
| Diouf et al. 2015 |  |  |  |  |  |  |  |  |  |  |  |  |  |  | x |  |  |  |  |
| Kamak et al. 2015 |  |  |  |  |  |  |  |  |  |  |  |  |  |  |  |  |  |  |  |
| Lee et al. 2015 |  |  |  |  |  |  |  |  |  |  |  |  |  |  |  | x |  | x | x |
| Liljestrand et al. 2015 |  |  |  |  |  |  |  |  |  |  |  |  |  |  |  |  | x | x | x |
| Yu et al. 2015 |  |  |  |  |  |  |  |  |  |  |  | x |  |  |  | x | x | x | x |
| Ahn et al. 2016 |  |  |  |  |  |  |  |  |  |  |  |  | x | x |  |  |  |  |  |
| Chen et al. 2016 |  |  |  |  |  |  |  |  |  |  |  |  |  |  |  | x |  |  |  |
| Fröhlich et al. 2016 |  |  |  |  |  |  |  |  |  |  |  |  |  | x |  |  |  |  |  |
| Górski et al. 2016 |  |  |  |  |  |  |  |  |  |  |  |  |  |  |  |  |  |  | x |
| Hansen et al. 2016 |  |  |  |  |  |  |  |  |  |  |  |  |  | x |  | x |  | x | x |
| Joshy et al. 2016 |  |  |  |  |  |  |  |  |  |  |  |  |  |  |  | x |  |  |  |
| Khatri et al. 2016 |  |  |  |  |  |  |  |  |  |  |  |  |  | x |  |  |  |  |  |
| Leira et al. 2016 |  |  |  |  |  |  |  |  |  |  |  |  |  |  | x |  |  |  |  |
| Rydén et al. 2016 |  |  |  |  |  |  |  |  |  |  |  | x |  |  |  |  |  |  | x |
| Tezuka et al. 2016 |  |  |  |  |  |  |  |  |  |  |  |  |  | x |  |  |  |  |  |
| Aoyama et al. 2017 |  |  |  |  |  |  |  |  |  |  |  |  | x |  |  |  |  |  |  |
| Çalapkorur et al. 2017 |  |  |  |  |  |  |  |  |  |  |  |  | x |  |  |  |  |  |  |
| Holmlund et al. 2017 |  |  |  |  |  |  |  |  |  |  |  |  |  | x |  | x |  | x | x |
| LaMonte et al. 2017 |  |  |  |  |  |  |  |  |  |  |  |  |  |  |  | x |  |  |  |
| Lee et al. 2017 |  |  |  |  |  |  |  |  |  |  |  |  |  |  |  | x |  |  | x |
| Sen et al. 2017 |  |  |  |  |  |  |  |  |  |  |  |  |  | x |  |  |  |  |  |
| Batty et al. 2018 |  |  |  |  |  |  |  |  |  |  |  |  |  |  |  | x |  |  |  |
| Kure et al. 2018 |  |  |  |  |  |  |  |  |  |  |  |  | x |  |  |  |  |  |  |
| Liljestrand et al. 2018 |  |  |  |  |  |  |  |  |  |  |  |  |  | x |  |  |  |  |  |
| Lysek et al. 2018 |  |  |  |  |  |  |  |  |  |  |  |  |  | x |  |  |  |  |  |
| Sen et al. 2018 |  |  |  |  |  |  |  |  |  |  |  |  |  |  | x | x |  |  |  |
| Khouja et al. 2019 |  |  |  |  |  |  |  |  |  |  |  |  |  | x |  |  |  |  |  |
| Lee et al. 2019 |  |  |  |  |  |  |  |  |  |  |  |  |  |  |  |  |  | x | x |
| Lin et al. 2019 |  |  |  |  |  |  |  |  |  |  |  |  |  |  |  | x |  |  |  |
| Niswade et al. 2019 |  |  |  |  |  |  |  |  |  |  |  |  |  | x |  |  |  |  |  |
| Wojtkowska et al. 2021 |  |  |  |  |  |  |  |  |  |  |  |  |  |  |  |  |  |  | x |

**Online Appendix S4**

Detailed analysis on the different outcome aspetcs.

1. CVD

A first SR (II) aimed to conduct a meta-analysis on observational studies that investigate the association between PerioD exposure and the risk of CVD in a large population sample. The researchers collected studies published between 1989 and 2007 from seven databases, resulting in 1413 references. Out of these, 25 cross-sectional or case-control studies and seven cohort studies were included in the analysis. The studies were conducted in various regions, with 11 among Scandinavian populations, eight in North America, eight in Europe, four in South America, and one in China. Although gender distribution was almost equal in cross-sectional and case-control studies, more women (58%) participated in cohort studies. The mean age was similar in both types of studies, with a baseline mean age of 59.3 years for cross-sectional and case-control studies and 52 years for cohorts. The SIGN^32^, Cho^33^ and Université Paul Sabatier (UPS)^34^ checklists were used to rate the overall quality of the included studies. Cross-sectional and case-control studies were found to be heterogeneous, with only eight studies validating internal, external, and ethical qualities. Cohort studies, on the other hand, were more homogeneous, with three studies validating both internal and external/ethical qualities and four validating only internal quality. Nine studies failed to validate internal quality. After excluding three studies with severe statistical heterogeneity, the random effects meta-analysis of 22 case-control and cross-sectional studies showed higher odds of developing CVD in patients with PerioD compared to those without (OR=2.35, 95%CI: 1.87-2.96, p<0.0001). Another fixed effects meta-analysis of seven cohort studies showed a 34% increased risk of developing CVD in patients with PerioD compared to those without (RR=1.34, 95%CI: 1.27-1.42, p<0.0001). Out of 34 results, 24 were found to be significant with OR or RR greater than or equal to 1.00. Based on the consistent and significant findings of the included studies, the review authors concluded that individuals exposed to PerioD had a greater likelihood and risk of getting CVD than individuals without PerioD.

The purpose of another study (X) was to conduct a SR and meta-analysis to investigate the risk of incident CVD outcomes in individuals with PerioD compared to those without. An electronic search was performed for multiple databases using relevant keywords and MeSH headings, with CVD outcomes including but not limited to MI, CHD, and stroke. After screening 1563 studies, 129 were fully reviewed and 32 were ultimately included in the meta-analysis. Additionally, meta-regression was used to evaluate the impact of key risk factors. Out of the 32 studies, 22 were prospective cohort studies, and 21 retrospective cohorts with individuals clinically diagnosed with PerioD. Of the studies that examined the risk of CVD, 14 focused on men and six on women. The majority of the studies included in the SR were conducted in the United States and had a median follow-up period of 14.5 years, with a range of 10.0 to 20.3 years. All studies reported age-adjusted risk, but five did not account for smoking. In terms of the ROBINS-I^35^ assessment, 21 of the studies were determined to have a critical risk of bias, while the other 11 were considered to have a serious risk of bias. The random effects meta-analysis showed a significant increase in the risk of all incident CVD in the PerioD population compared to the non-PerioD population, with an RR of 1.20 (95%CI: 1.14-1.28). Heterogeneity was high due to the large sample size in most studies (I^2^=98.1%). After conducting a sensitivity analysis by excluding five studies that did not adjust for smoking, the risk of CVD remained unchanged. Based on these findings, the review authors indicated that individuals with PerioD have a higher risk of developing CVD.

1.1 Cerebrovascular disease

The primary objective of the SR (VIII) was to analyze observational studies that explored the relationship between PerioD and CHD and cerebrovascular diseases. The search yielded 292 titles and abstracts. Ultimately, seven cohort studies and four cross-sectional and retrospective studies were selected. The cohort studies included in this analysis had a range of participants, from 1147 to 44119, and spanned an age range of 25 to 84 years. The cases were defined as individuals who had either fatal or non-fatal CHD and cerebrovascular disease. A separate analysis was performed to evaluate the association between PerioD and cerebrovascular disease including four cohort studies, one cross-sectional study, and one case-control. Among these, only three studies demonstrated a statistically significant association. Overall, the RR of cerebrovascular disease for individuals with PerioD was 1.13 (95%CI: 1.01-1.27; p=0.032) compared to healthy individuals. However, when the analysis was limited to cohort studies only, the association between PerioD and cerebrovascular disease risk was no longer statistically significant (RR=1.11, 95%CI: 0.98-1.25). The studies showed no statistical heterogeneity in the overall relative risk estimate (p=0.472). As suggested by the review authors, the results of this meta-analysis should be interpreted with caution as it is based on observational studies, which may have biases and confounding factors that could affect the accuracy of the findings. Although meta-analysis of observational studies is useful in understanding variability across studies, it can also present challenges due to inherent biases and differences in study designs. There is also a possibility of publication bias, although the analysis did not indicate any evidence of it. According to the authors of the review, it is important to be cautious when interpreting data from this meta-analysis and avoid relying solely on the statistical significance testing.

1.2 CHD

One SR (I) performed a meta-analysis with the aim to establish a correlation between PerioD and CHD. The search identified a total of 320 papers, of which 20 were eligible for inclusion in the meta-analysis. Only 15 of the 20 eligible studies were used for quantitative meta-analysis due to the heterogeneity between studies in the type of risk estimates and the lack of basic data to compute uniform risk estimates. Only studies that adjusted for confounding factors were included, which improves the quality and validity of the meta-analysis. The 15 studies used included five prospective cohort studies reporting RR (follow-up > 6 years), 5 case-control studies, and 5 cross-sectional studies, all of which reported OR. The review authors analyzed the study categories separately. A meta-analysis of five prospective cohort studies, involving a total of 86092 patients, showed that individuals with PerioD had a 1.14 times higher risk of CHD compared to healthy individuals (RR=1.14, 95%CI: 1.074-1.213, p<0.001). An even greater risk of CHD was found in case-control studies, involving 1423 patients (OR=2.22, 95%CI: 1.59-3.117, p<0.001). Among the cross-sectional studies, involving 17724 patients, a significantly higher prevalence of CHD was found for individuals with PerioD compared to those without PerioD (OR=1.59, 95%CI: 1.329-1.907, p<0.001). The review authors suggests that there may be a link between PerioD and CHD based on the evidence of increased incidence and prevalence of CHD in individuals with PerioD.

A more recent review (V) aimed to evaluate the occurrence of CHD or fatal CHD analyzing studies using PerioD or number of teeth as their exposure variable. Fatal CHD events were defined as the incidence of MI and sudden death without any plausible causes other than CHD. The search identified 877 unique titles and abstracts of which 11 cohort studies met the eligibility criteria. A meta-analysis of 10 studies was performed to analyze the relationship between PerioD and CHD, revealing that patients with PerioD had a greater risk of developing CHD compared to those without PerioD (OR=1.18, 95%CI: 1.10-1.26). Only one study reported no association between PerioD and CHD. The review authors found a significant increased risk for CHD in individuals with PerioD and therefore concluded that there is a meaningful as­sociation between the two diseases.

Study VI performed a meta-analysis to determine the bidirectional causality between PerioD and CHD. The database search yielded 143 titles and abstracts resulting in seven eligible cohort studies, which span cohorts of 175–170000 men and women with follow-up ranging from 5–21 years. The studies were carried out in North America and Finland. Since the reported CHD outcomes among the included studies differed, the CHV outcomes were divided into four groups: CHD events (including MI, coronary admission, or revascularization), deaths due to CHD, CVD/CVE (including stroke, and PVD), and deaths due to cardiovascular diseases. The association between PerioD at baseline and the risk of CHD varied across the six cohorts examined in the review. While three cohorts showed a significant positive association, three others did not. However, when the studies were pooled together in a meta-analysis, the overall estimate of RR for any CHD or CVD/CVE was found to be 1.24 (95%CI: 1.01–1.51). Nevertheless, significant heterogeneity was observed among the studies, indicating the need for caution in interpreting the findings. The review authors suggest that if there is no causal relationship between PerioD and CHD, it could be considered as a risk marker. According to their theory, there can be unexplained confounding brought on by a component that is linked to both PerioD and CHD. Some known factors that may contribute to this include smoking, diet, diabetes, and socio-economic status. However, despite adjustment for all these factors in almost all studies included in the review, an increased risk was still identified.

A fourth SR (VII) quantitative summaries studies determining the possible association between PerioD and CHD and stroke. A comprehensive search was conducted in the MEDLINE-PubMed database to identify all published articles that examined the relationship between PerioD as the predictor variable and outcomes such as CHD, stroke, or both. Nine cohort studies were included, of which eight prospective and one retrospective involving 107011 individuals and 7035 CVE. The meta-analysis showed that individuals with PerioD have 1.19 (95%CI: 1.08-1.32), more risk of developing CHD compared to individuals without PerioD. The results of the meta-regression analysis were affected by both overestimation (12.9%) and underestimation due to residual confounding and the use of a proxy (29.7%), respectively and significant heterogeneity (p=0.003) was found. The review authors conclude that there may be a significant link between PerioD and cardiovascular diseases, with the risk of developing cardiovascular diseases in the general population being around 20% between individuals with and without PerioD. Suggesting that the finding may have significant impact on public health, since almost 40% of the population has PerioD.

One other SR (VIII) explores the relationship between PerioD and CHD and cerebrovascular diseases. A meta-analysis of eight studies, six cohort studies and two cross-sectional studies was performed to analyze the association between PerioD and total CHD. The studies included in the meta-analysis consistently showed that individuals with PerioD had a higher risk of developing CHD compared to healthy individuals, even after accounting for potential confounding factors. However, this difference was only statistically significant in two of the cohort studies, with RR ranging from 1.25 to 1.50. The overall adjusted risk of CHD in subjects with PerioD compare to individuals without PerioD was found to be 1.15 (95%CI: 1.06-1.25; p=0.001). Although there was no significant statistical heterogeneity in the overall risk ratio estimation, the review authors still consider the possibility of substantial differences between the studies. Additionally, when only analyzing prospective studies or when excluding the studies relying on self-reported exposure, the overall RR was slightly decreased in both cases to 1.14 (95%CI: 1.04-1.25; p=0.004). Conversely, a significant correlation between PerioD and fatal CHD was not found, as the RR was 1.20 (95%CI: 0.90-1.60; p=0.205). The review authors suggest that the reported positive associations may have been influenced by small biases or residual confounding, as both PerioD and CHD share several common risk factors, such as advancing age, smoking, stress, socioeconomic status, body fat content, and health consciousness. Based on these findings, the review authors conclude that it remains uncertain whether the correlation between oral health and heart disease is specific or merely coincidental.

A sixth study (X) was conducted to investigate the risk of developing CVD/CVE in individuals with PerioD compared to those without PerioD. An electronic search across multiple databases was performed focusing on cardiovascular diseases outcomes such as MI, CHD, and stroke. After screening 1563 studies, 129 studies were fully reviewed and ultimately 32 studies were included in the meta-analysis. Additionally, meta-regression was used to assess the influence of key risk factors. Among the 32 included studies, 13 were included in a separate meta-analysis to analyze the effect of PerioD on CHD showing that the risk of CHD increased by 14% (RR=1.14, 95%CI: 1.08-1.21) in patients with PerioD compared to those without. High heterogeneity was observed due to the large sample sizes in most studies (I^2^=98.1%). Based on these findings, the authors of the review concluded that individuals with PerioD have a significantly higher risk of developing cardiovascular diseases, particularly CHD.

Study XII systemically reviews the effect of PerioD on acute coronary syndrome (ACS). ACS is a collective term encompassing various conditions, such MI and unstable angina, all of which fall under the category of CHD.^36^ The initial search yielded 254 studies with identical titles and abstracts. After applying the inclusion and exclusion criteria, a total of 46 studies were included, involving 6806286 participants and 68932 cases of ACS. These studies encompassed various research designs, including cohort, cross-sectional, and case-control. Only studies that reported adjusted effect sizes were included in the analysis. The results of the meta-analysis demonstrated a significant association between PerioD and a higher risk of ACS (OR=1.35, 95%CI: 1.25-1.45). However, statistical heterogeneity was observed among the included studies (I^2^=86%, p<0.05). Subgroup analyses based on study design revealed significant differences. Notably, case-control studies exhibited the strongest association between PerioD and ACS (OR=2.62, 95%CI: 2.05-3.35), followed by cohort studies (OR=1.13, 95%CI: 1.05-1.21). The association between PerioD and ACS was not found to be significant in cross-sectional studies (OR=1.67, 95%CI: 0.79-3.50). To assess the risk of bias in the included studies, the Newcastle-Ottawa Scale (NOS)^37^ was employed. As a result, four studies were identified as having a high risk of bias, while the remaining studies were identified to have a low risk of bias. Additionally, significant publication bias was detected, indicating that the literature may be biased towards reporting positive results. Based on this meta-analysis, the authors of the review concluded that PerioD may be considered a non-traditional risk factor for developing ACS.

One other descriptive analysis (XVI) examined the link between PerioD and IHD exclusively. A total of 890 entries related to the relationship between PerioD and cardiovascular diseases were found in three databases limited from 2014 till 2019. Eventually, 17 references met the inclusion criteria, including three case-control studies, four cross-sectional studies, six prospective cohort studies, and four retrospective cohort studies. The sample size varied from under 100 persons to over 10000 individuals, with age and sex inconsistently specified. Only two studies provided race-related information. Results were reported to a control group in only 40% of studies, and the follow-up period ranged from 1-33 years. The selected studies aimed to investigate the links between PerioD and various CVD/CVE, including chronic heart failure, MI, stroke, as well as endothelial function. According to their review, strong evidence for a relationship between PerioD and IHD is found. However, it should be noted that the methodological heterogeneity among the studies was reported as high by the review authors. Based on the findings of this qualitative analysis, the review authors concluded that there is a association between PerioD and CVD/CVE but the causal mechanism underlying the link is not yet well-established.

1.3 ACVD

A first study (III) aimed to conduct a SR of the epidemiological evidence regarding the potential association between PerioD and the incidence of ACVD, which includes CHD, cerebrovascular disease, and peripheral arterial disease (PAD). The analyzed studies focused on the occurrence of several health conditions, including angina, MI, CHD-related death, as well as transient ischemic attack, stroke, and PAD. The electronic literature search yielded 1395 studies. After the screening and selection process 12 studies remained eligible for inclusion. Due to the substantial variability observed among the studies in terms of numerous factors, such as study populations, methods used to assess and define exposure and outcomes, and the way in which potential confounders were identified and statistically adjusted for, a meta-analysis was not performed. Furthermore, there were multiple study reports that utilized the same population of participants but focused on various ACVD outcomes. Specifically, data from the Department of Veterans Affairs Normative Ageing and Dental Longitudinal Studies, which were conducted in Boston, MA, USA, were presented in separate publications pertaining to CHD, cerebrovascular disease, and PAD. The qualitative analysis included three cohort studies and three case-control studies that specifically focused on CHD, one cohort study and two case-control studies that concentrated exclusively on cerebrovascular disease and one cohort study that exclusively addressed PAD. Additionally, two more cohort studies were identified that examined ACVD mortality, encompassing both CHD and cerebrovascular disease as potential causes of death. Except for one study, all the identified studies reported a positive association between PerioD, and the development of ACVD, independently of established cardiovascular risk factors. However, as stated by the review authors the available evidence concerning a potential association between PerioD and PAD was limited, with only one study addressing these outcomes.

The purpose of a second review (XIII) was to investigate the association between PerioD and vascular alterations through a meta-analysis of current data. The review focused on carotid intima-media thickness (c-IMT) and flow-mediated dilatation (FMD) as markers of ACVD. C-IMT is a non-invasive technique that has been found to be correlated with an individual's cardiovascular risk factor burden and the risk of future CVE, such as coronary artery disease and stroke. FMD is used to assess endothelial dysfunction, which occurs in the early stages of ACVD. A total of 2066 hits were obtained from electronic searches, and 101 publications were selected for full-text review, resulting in 35 eligible studies for inclusion in the SR. Out of the 35 studies, 22 were included in the meta-analyses. All studies reported evidence on the association between cardiovascular diseases markers and PerioD. However, there was considerable variation in terms of PerioD definition, population, sample size, age range, carotid segments studied, and definition of c-IMT among the included studies. Eventually, 16 studies were included for further analysis, as ten did not provide comparable estimates. According to the NOS,^37^ the majority of studies were categorized as having a low or medium risk of bias, with some discrepancies in periodontal diagnoses, definition of controls, and adjustment for confounders. Furthermore, methodological heterogeneity was identified, particularly in the definition of carotid segments. The meta-analysis showed a significant difference for both c-IMT (p<0.001) and FMD (p<0.001) for patients with PerioD compared to those without. Based on the outcome of this review, the review authors concluded that there is an increased risk of subclinical ACVD for individuals with PerioD.

One other SR (XVII) aimed to determine, based on the available published evidence, the association between PerioD and the incidence of peripheral artery disease (LEAD) or coronary atherosclerosis (CAD). The purpose of this review was to explore the relationship between PerioD and PAD, as well as to identify potential risk factors for PerioD. The search yielded 3543 titles and abstracts. After applying the inclusion and exclusion criteria a total of 25 studies were included with a total of 22090 subjects. The studies were conducted in four continents and 14 countries, with a mix of cohort, cross-sectional and case-control designs. The assessment of LEAD and CAD was commonly done using ankle brachial index (ABI) or c-IMT. Three meta-analyses were conducted in this SR of which 25 studies investigated the link between PerioD and PAD, 16 studies investigated the link between PerioD and incidence of CAD, and nine studies investigated the association between PerioD and LEAD. However, the definition of PerioD differed across the various studies. The quality of the studies was assessed using the NOS,^37^ and 16 studies were considered of high quality. Factors such as age, smoking, and metabolic diseases were adjusted for in some studies. Out of the 25 studies included in the analysis, only three studies reported a negative association between PerioD and PAD, while the majority of studies suggested a consistent and significant positive correlation. The studies included in the meta-analysis conducted to analyze the incidence of CAD in PerioD patients showed significant heterogeneity (I^2^=79.4%; p<0.001), but in general, the cohort with PerioD had a higher association with CAD compared to the reference group (OR=1.39, 95%CI: 1.24-1.56; p<0.001). Based on this meta-analysis, the review authors concluded that there is a significant association between PerioD and CAD.

Study XIX conducted a SR with the purpose to assess the correlation between PerioD and CAD. A systematic literature search was conducted and identified 273 potential articles. After further assessment, 15 observational studies were included in the meta-analysis. All included studies were either case-control or cross-sectional studies. The reference group varied among the studies, with 10 studies enrolling participants without PerioD and five using patients with mild or no PerioD. The results of the sub-analysis focused on studies that included participants without PerioD suggests that individuals with PerioD may be at a higher risk of developing CAD (OR=1.75, 95%CI: 1.33-2.30; p<0.01). The association was found to be significant even after adjusting for other potential confounding factors, such as smoking and DM. Based on these findings, the authors of the review suggested that PerioD may be an independent risk factor for CAD.

1.4 PVD

Study XVII assessed the link between PerioD and PAD based on recent available data. The primary objective of this review was to evaluate the association between PerioD and the incidence of LEAD and CAD. With a total of 22090 subjects, three meta-analyses were conducted of which 25 studies investigated the link between PerioD and PAD, 16 studies investigated the link between PerioD and incidence of a CAD, and 9 studies that investigated the association between PerioD and LEAD. A negative association between PerioD and PAD was reported in three of the included. A meta-analysis was performed and showed that individuals with PerioD have a higher risk of developing PAD compared to those without the disease (OR=1.60, 95%CI: 1.41-1.82; p<0.001), even after adjusting for confounding factors. Based on these finding, the authors suggested that PerioD could possibly be an independent risk factor for PAD. Furthermore, the meta-analysis of the subgroup confirmed the review authors conclusion by revealing a significant increase in the risk of developing LEAD among people with PerioD compared to the reference group (OR=3.00, 95%CI: 2.23-4.04; p<0.001). This meta-analysis showed a significant association between PerioD and an elevated risk of PAD, including both LEAD and CAD. For the increased incidence of both LEAD and CAD, the review authors stated that PerioD is an independent risk factor for developing PAD.

2. CVE

2.1 Stroke

A first SR (IV) and meta-analysis purposed to examine the correlation between PerioD and stroke, analyzing various forms of cerebrovascular diseases. The electronic database search yielded 2193 studies and after removing duplicates 1235 studies remained for the screening and selection process. Eventually, this study analyzed seven case-control studies (with a range of 80-606 participants) and three cohort studies (with a range of 7674-10362 participants). The cohort studies had a follow-up time ranging from 12-21 years. Case-control and cohort studies were analyzed separately, and another distinction was made between ischemic stroke and stroke. The authors of the review determined the level of evidence of the included studies, using the GRADE^38^ approach. Overall, the evidence for the association between PerioD and stroke was limited due to various methodological issues in the included studies. The first meta-analysis included seven case-control studies and found that individuals with PerioD had a two-fold higher chance of suffering from stroke (OR=2.31, 95%Cl: 1.39-3.84; p=0.001). The second meta-analysis focused on five case-control studies that evaluated ischemic stroke and found a similar two-fold higher chance in individuals with PerioD (OR=2.72, 95%CI: 2.00-3.71; p<0.00001). The third meta-analysis analyzed three cohort studies and found that individuals with PerioD had a higher risk of suffering from stroke (RR=1.88, 95%CI: 1.55-2.28; p<0.00001). Based on these findings, the authors of this SR gathered scientific proof that there is a link between PerioD and several forms of stroke. According to the meta-analysis's results, those with PerioD had a about twice as high risk of experiencing any sort of stroke. However, the results from the included studies were inconsistent. Although the majority of the papers qualitatively discussed a link between PerioD and stroke, this association was only found in cohort studies and in case-control studies for ischemic stroke events. Furthermore, the review authors suggested that these findings are questionable because of the significant degree of heterogeneity among the included studies.

A second SR (VII) evaluates the link between PerioD, CHD and stroke. This meta-analysis incorporated nine cohort studies, out of which eight were prospective and one was retrospective. The study analyzed a total of 107011 individuals and 7035 CVE. A separate meta-analysis was conducted for both stroke and fatal cardiac diseases. The analysis showed that for fatal cardiac diseases and stroke outcomes, the RR were 1.54 and 2.85, respectively. The pooled estimate of the RR of stroke outcomes in relation to PerioD was found to be 2.85 (95%CI: 1.78-4.56, p=0.000), based on two studies included in the analysis. Nonetheless, inadequate adjustment for confounding factors in certain studies led to an overestimation of the RR by 12.9%, while the use of questionnaires resulted in an underestimation of the RR by 29.7%. Taking these factors into account, the review authors stated that the overall risk increase may still be lower than the true RR.

The objective of one other review (IX) was to use a meta-analysis of cohort studies to investigate the relationship between PerioD and stroke incidence. The inclusion criteria required that the studies evaluated stroke incidence (fatal or non-fatal, ischemic, or hemorrhagic), measured baseline periodontal status, and calculated RR values. Nine cohort studies out of the 743 found references were eligible for analysis. Five studies were conducted in North America between 1970-1980, with participant numbers ranging from 1137-51529 and follow-up periods ranging from 12-57 years. The studies were assessed for quality using the National Health and Medical Research Council (NHMRC)^39^ evaluation grid. The highest score that could be obtained was 16. For the included studies the quality scores ranged from 7-13, with four studies scoring 12 or more. The highest scores were obtained by three prospective studies that used reliable indicators of PerioD and took into account major confounding factors. Five studies included all confounding risk factors in the calculation of adjusted risk values, and in some studies, the patients' previous history of stroke was not considered. Smoking was the only factor considered in all studies, and heterogeneity in the indicators used to evaluate PerioD introduced a major bias, with clinical oral examination conducted in seven studies and self-evaluation through a questionnaire in two studies. The analysis was performed separately for three outcomes: PerioD, gingivitis, and TL. Another separate meta-analysis of cohort studies was performed, which had varying quality scores, to obtain pooled data. The analysis found an association between stroke and PerioD, with PerioD and TL emerging as important risk factors. The adjusted risk estimates showed that individuals with PerioD had a higher risk of both ischemic and hemorrhagic stroke, with the estimated risk being 1.63 (RR=1.63, 95%CI: 1.25- 2.00). Based on the findings of this qualitative analysis, the authors concluded that there is a link between PerioD and stroke.

One study (X) aimed to systematically review the existing literature to examine the association between PerioD and incident cardiovascular diseases outcomes, such as MI, CHD, and stroke. The electronic database search yielded 1563 studies and after the screening and selection process 32 were included for further analysis. Among individual CVD/CVE outcomes 19 studies evaluated the incidence of stroke and six studies evaluated the incidence of MI among patients with and without PerioD. A separate meta-analysis was conducted for both showing a 24% higher incidence of stroke for individuals with PerioD compared to those without (RR=1.24, 95%CI: 1.12-1.38). After conducting a sensitivity analysis by excluding five studies that did not adjust for smoking, the risk of cardiovascular diseases remained unchanged, with the risk of stroke being the highest. Based on these findings, the review authors indicated that individuals with PerioD have a higher risk of developing CVD/CVE.

A fifth SR (XI) performed a meta-analysis to determine whether there is a connection between PerioD and stroke and, if so, how important it is. The objective was to offer a SR and meta-analysis of the available data examining the relationship between PerioD and ischemic stroke. The search in multiple databases yielded 414 titles and abstracts and after screening 57 possibly pertinent full text papers were found. After applying inclusion criteria, eight studies were eligible for analysis. The included studies were conducted in five different countries across Europe, North America, and Asia, published between 1996 and 2014. The population sample size ranged from 95-9962 patients, with three studies being cohort studies and five being case-control studies. Based on the GRADE^38^ checklist, four studies were of low quality while the remaining studies were of high quality, all of which were included to achieve the goal of the SR. Heterogeneity was found high for all studies, but it decreased when studies were stratified by design, while the pooled estimate remained significant. However, in case-control studies, the heterogeneity remained high even after stratification. Further analysis showed that high-quality studies found a strong association between PerioD and stroke with less heterogeneity, while low-quality studies showed a weaker but still significant effects with high heterogeneity. Based on the findings of the cohort studies, a positive association between the prevalence of PerioD and ischemic stroke is found, indicating that individuals with PerioD are at a higher risk of cerebral ischemia.

One other SR (XV) had the objective to investigate the potential link between PerioD and cerebrovascular disease in observational studies. The authors aimed to obtain a summary of the combined findings on the relationship between PerioD and the occurrence of cerebrovascular disease. At the beginning of the search, 146 abstracts were identified. Eventually, 13 studies met the inclusion criteria and were included in the analysis, consisting of six prospective studies and seven retrospective studies. If a study categorized PerioD severity into more than two groups, only the least and most severe categories were used for the analysis. The studies that were included in the analysis examined various outcomes related to stroke, including non-hemorrhagic and hemorrhagic stroke, nonfatal stroke, ischemic stroke, stroke death (hemorrhagic and ischemic), as well as ischemic stroke or transient ischemic attack (TIA). To ensure accuracy, separate analyses were conducted for both prospective and retrospective studies. For this meta review only the results of the prospective studies were evaluated since the other studies in this SR used PerioD as outcome instead of exposure variable. The included prospective studies had a number of participants ranging from 1137-44119 aged 25-84 years old. All studies adjusted for potential confounding variables, with age, hypertension, smoking, DM, hyperlipidemia, and body mass index being the most adjusted factors. In all studies, the RR was greater than one. Among the six eligible prospective studies analyzed, five showed a statistically significant association (p<0.05). The findings of the meta-analysis indicate that individuals with PerioD have a 1.47-fold higher adjusted risk of stroke compared to those without PerioD (95%CI: 1.13-1.92; p=0.0036). Significant heterogeneity was found among all studies. However, sensitivity analysis did not reveal any significant differences in the findings. Although the possibility of publication bias cannot be ruled out, the trim and fill correction resulted in changes to the estimates that reduced the impact of PerioD on stroke risk without altering the overall conclusion. This meta-analysis indicates that there is a significant association between PerioD and stroke and even after accounting for other potential risk factors, socioeconomic variables, and lifestyle factors this association remains.

2.2 MI

One study (X) performed a meta-analyses to analyze the link between PerioD and CVD/CVE. The authors of the review further divided cardiovascular diseases into MI, CHD, and stroke. An electronic database search was performed followed by screening and selection procedures, eventually resulting in 32 included studies. Distinct meta-analyses were conducted for each cardiovascular event as 19 studies specifically examined the incidence of stroke, while six studies focused on the occurrence of MI in individuals with and without PerioD. The meta-analysis showed an RR of 1.12 (95%CI: 0.96-1.30) for MI in patients with PerioD compared to those without. The review authors concluded that individuals with PerioD have a higher risk of developing CVD/CVE, however the increased risk of MI was not significant based on the 95% confidence interval bounds.

A second SR (XIV) aimed to investigate the association between PerioD and the risk of MI. The researchers conducted a SR and meta-analysis of cohort studies that examined the potential link between PerioD and MI. An electronic search in three databases identified 1155 potential results and after screening titles and abstracts 61 full papers were obtained. Eventually 10 cohort studies, with a publication date ranging from 1996-2019, were eligible for inclusion. The included studies involved a total of 5369235 participants aged 20 or older. The follow-up period of the studies varied from 5-3 years and of the 10 studies, four were conducted in the USA, three in European countries, and three in Asian countries. All studies adjusted for potential confounding factors. Four of the studies did not find a significant association between PerioD and MI, while six studies found a positive correlation between the two diseases. According to the NOS,^37^ five studies were of moderate quality, and the other five studies were of high quality. Due to significant statistical heterogeneity among the studies (I^2^=78.0%), a random effects model was utilized for this meta-analysis. The meta-analysis of the cohort indicated a significant association between PerioD and MI (OR=1.13, 95%CI: 1.04–1.21; p=0.004). When dividing the studies by quality assessed by the NOS,^37^ conflicting results were found. The high-quality group showed an association between PerioD and MI (OR=1.20, 95%CI: 1.03–1.37; p=0.019), while the moderate quality group showed no increase (OR=1.09, 95%CI: 0.94–1.24; p=0.254). Based on the meta-analysis, the review authors concluded that there is a slight association between PerioD and the risk of MI.

Study XVIII evaluated the relationship between PerioD and MI by conducting a meta-analysis of observational studies. The database search yielded 2558 and after the evaluation of titles and abstracts 373 studies were further evaluated. After reading full texts, 22 studies met the inclusion criteria and were included in this meta-analysis, representing 129630 participants. Heterogeneity was observed in all three types of studies, with the highest level in case-control studies. For this meta review only the results of the cohort studies were evaluated since the other studies in this SR used PerioD as outcome instead of exposure variable**.** The cohort studies were all conducted in America and the enrollment years varied from 1996 to 2015. In three of the four cohort studies, no significant association between PerioD and the risk of MI was found. A subgroup meta-analyses was performed by study type and showed that, in cohort studies, a marginally, but not significant, increased risk for MI was found (OR=1.18, 95%CI: 0.98-1.42). While the association in the cohort studies was weak, the review authors found an overall statistically significant association between PerioD and MI, with subgroup analyses further confirming the increased risk of MI in individuals with PerioD.

3. Subgroup analysis

3.1 Gender

One descriptive analysis (III) states that there are various studies that examine the effect of gender on the association between PerioD and CVD/CVE, but the findings are not consistent. Some studies suggest that the association is stronger in men than women for cardiovascular diseases and both CHD and cerebrovascular disease. In contrast, one study found a stronger association in women than men for CHD, while another study found no association between PerioD and CHD for gender.

One other SR (VI) performed a meta-analysis showing **t**he risk estimates for CHD or CVD/CVE events by gender variability. A higher risk among women with PerioD (RR=1.59, 95%CI: 1.28-1.96) was observed compared to men (RR=1.23, 95%CI: 0.92-1.64), but the differences were not statistically significant.

In the subgroup analysis stratified by gender of study XVIII, a significant elevated risk of MI in the female pooled studies was found (OR=1.64, 95%CI: 1.20-2.25). the pooled results from the male studies showed a marginal increase (OR=1.18, 95%CI: 0.96-1.44), but not statistically significant. The subgroup analyses conducted based on various factors indicate that individuals with PerioD are at a greater risk of developing MI.

One SR (X) showed that men with PerioD have a 16% higher incident risk of cardiovascular diseases (RR=1.16, 95%CI: 1.08–1.25) compared to men without PerioD, while women with PerioD have an 11% higher risk (RR=1.11, 95%CI: 1.02–1.22). However, there was no significant difference in cardiovascular diseases risk between genders (RR=1.04, 95%CI: 0.92–1.17). The authors of this SR concluded that individuals with PerioD have a high risk of developing CVD/CVE, with men having an even higher risk.

Study XII performed a subgroup analysis for gender and found a higher risk of ACS in women with PerioD (OR=1.96, 95%CI: 0.62-6.17), compared to men with PerioD (OR=1.48, 95%CI: 1.11-1.97). However, the difference was not statistically significant.

In one other analysis of subgroups by gender (XIV), the combined findings of three studies among males did not show any correlation between PerioD and MI (OR=1.05, 95%CI: 0.89–1.24; p=0.560). However, one study including females reported an elevated risk of MI (OR=1.39, 95%CI: 1.17–1.65; p=0.000) ^40^. This meta-analysis of cohort studies suggests that there is a slight connection between PerioD and the risk of MI, particularly in women.

3.2 Age

A first SR (II) performed of meta-regression and subgroup analyses showing that the effects of mean age were either non-significant or borderline. Specifically, the p-value for cross-sectional/case control studies was 0.11, and for cohort studies, it was 0.06.

One other SR (III) evaluated the effect of PerioD on CHD by conducting a descriptive analysis noting that various studies have reported subgroup analyses by age, with different age cut-offs ranging from 60 to 65 years. Younger subjects were found to have stronger associations between PerioD and CHD incidence compared to older subjects. In addition, there was no indication of an association between PerioD and incident CHD in individuals aged ≥ 65 years old.

A meta-analysis of nine studies (VII) showed that patients with PerioD had a 1.19 (95%CI: 1.08-1.32) increased risk of CHD. This risk further increased to 1.44 (95%CI: 1.20-1.73) in individuals aged ≤ 65 years old.

3.3 PerioD severity

A first SR (III) qualitative evaluated the available evidence and indicates that individuals with more severe PerioD and/or poorer periodontal health have a higher incidence of ACVD, including CHD, cerebrovascular disease, and PAD compared to no or less severe PerioD. This association persists even after adjusting for various established cardiovascular risk factors.

One other SR (X) found that the risk of developing incident CVD/CVE was increased with the severity of PerioD, with higher risks observed in moderate and severe PerioD compared to mild PerioD. Specifically, the risk was 9% for mild PerioD, 23% for moderate PerioD, and 25% for severe PerioD. The risk of developing incident CVD/CVE was significantly higher in severe PerioD compared to mild PerioD (RR=1.11, 95%CI: 1.00–1.22). Individuals with severe PerioD have the highest risk of developing cardiovascular diseases.

A third SR (XVI) described that severe PerioD has been found to be a significant risk factor for cardiac death, all-cause mortality, recurrent strokes, and vascular ACVD in the Korean population. The association between PerioD and IHD is stronger in patients with severe PerioD, indicating that it could be a potential risk factor for the disease. As such, PerioD should be taken into account in the management of CVD/CVE.

One other subgroup meta-analyses (XIX) was performed to evaluate the effect of PerioD severity for both moderate and severe PerioD. Based on four included studies patients with moderate PerioD were found to have a 1.10 (95%CI: 1.04–1.16; p<0.01) times higher risk of developing CAD. Severe PerioD was analyzed in five studies and associated with a 1.14 (95%CI: 1.06–1.23; p<0.01) times higher risk of CAD. Based on the results of the meta-analyses, the review authors conclude that there appears to be a direct association between the severity of PerioD and the risk of developing carotid atherosclerosis.

3.4 Smoking status

One SR (III) performed a qualitative analysis and evaluated one primary clinical study ^41^ that examined the effect of PerioD on CVD/CVE for smoking. This study discovered a notable association solely in individuals who had never smoked as compared to those that had smoked in the past.

The authors of a second descriptive review (XVI) concluded that there is evidence of an increased risk of CVD/CVE and acute cardiovascular diseases associated with PerioD among smokers. These findings were also based on one primary study.^42^

3.5 Study region

One SR (II) conducted a subgroup analyses and meta-regression based on the geographic area of origin. The results showed that there was a higher OR for incident cardiovascular diseases in people with PerioD from Europe (OR=3.95, 95%CI: 2.54-6.15), South America (OR=2.34, 95%CI: 1.48-3.70), North America (OR=2.02, 95%CI: 1.49-2.73), and Scandinavian countries (OR=2.00, 95%CI: 1.27-3.15), but the difference was not statistically significant (p=0.08). In addition, the geographic area of origin of the population in cohort studies had a potential influence on the global RR, with higher RR observed in North America (RR=1.50, 95%CI: 1.34-1.68), Scandinavian countries (RR=1.30, 95%CI: 1.17-1.44), and China (RR=1.28, 95%CI: 1.17-1.40), but the difference was not statistically significant (p=0.08).

One other study (X) found a higher incident risk of CVD/CVE (RR=1.36, 95%CI: 1.20–1.54) in European studies compared to studies from Asia/Australia by 18% (RR=1.18, 95%CI: 1.03–1.35). However, the incident risk of cardiovascular diseases in European studies was only 3% higher (RR=1.03, 95%CI: 0.93–1.13) compared to studies from North America.

Study XII found a statistically significant difference in risk of ACS between study regions. Specifically, studies conducted in South America demonstrated the strongest association (OR=4.43, 95%CI: 2.39-8.23). In Europe, a significant association was also observed (OR=1.92, 95%CI: 1.59 -2.31) and similarly for studies conducted in North America (OR=1.30, 95%CI: 1.16-1.46). However, the association between PerioD and ACS in studies conducted in Asia was not found to be statistically significant (OR=1.09, 95%CI: 0.96-1.25).

**Online Appendix S5**

Detailed analysis of the Bradford Hill criteria.^43^

- **Strength of association**: The results of almost all meta-analysis conducted in the SRs

showed significant associations for both pooled OR and pooled RR. However, most of the OR and RR were between 1.0 and 2.0, as shown in Table 3A, and therefore the strength of the association in general could be considered as weak.^44,45^ In five SRs (I, II, IV, XI and XV) a RR^44^ or OR^45^ was found to be meaningful (>2.0), of which three SRs specifically examined the association between PerioD and stroke (IV and XI, Table 3A).

- **Consistency:** All SRs showed a significant association between PerioD and CVD and stroke, but the relationship between PerioD and MI was less conclusive (Table 3A). However, studies show in general a negliable to small magnitude of the association.
- **Specifically:** The studies included in this synthesis of SRs did not report the same outcome in every instance.
- **Temporality:** Based on the included SRs, temporality could not be established.
- **Biological gradient:** As analyzed in four SRs (III, X, XVI and XIX, Table 3B), all showed a consistent increased risk of CVD and CVE in relation to the severity of PerioD.
- **Plausibility:** Inflammatory cytokines play a crucial role in both PerioD and CVD and CVE, and numerous studies have evidence supporting this association.^46–49^ Elevated levels of these cytokines have been observed in both conditions, suggesting shared mechanisms between PerioD and CVD/CVE.
- **Coherence:** Previous in vitro and in vivo studies, both in animals and humans, have established a relationship between PerioD and CVD and CVE.^50,51^ Additionally, other studies have linked PerioD with the development of other systemic diseases with conditions such as DM, pre-term birth and respiratory infections.^52–54^ However, this synthesis of SRs suggests a mostly negligible to small magnitude of association between PerioD and CVD/CVE, challenging the more assertive claims made in previous studies.
- **Experiment:** Although the specific criterion 'experiment' may not be directly applicable in the evaluation of the association between PerioD and CVD/CVE, this synthesis of SRs followed a rigorous approach by considering SRs that examined the association between PerioD and CVD/CVE parameters. This approach allowed for a thorough assessment of the available evidence from various study designs (cohort, cross-sectional, and case-control), which can contribute to understanding the potential causal relationship between PerioD and CVD/CVE.
- **Analogy:** The criteria ‘analogy’ was not explored in this synthesis of SRs since animal studies were excluded. While animal studies can provide insights into potential biological pathways and establish initial associations, the extrapolation of findings to human populations requires caution. This syntheses prioritized evidence derived from human studies to directly assess the association of PerioD and CVD/CVE parameters in individuals.

**Online Appendix S6**

List of abbreviations.

| **Abbreviation** | **Meaning** |
| --- | --- |
| ABI | Ankle brachial index |
| ACS | Acute coronary syndrome |
| ACTA | Academic Center for Dentistry Amsterdam |
| ACVD | Atherosclerotic cardiovascular disease |
| AF | Aterial fibrillation |
| CAC | Carotid artery calcification |
| CAD | Coronary atherosclerosis |
| CHD | Coronary heart disease |
| C-IMT | Carotid intima-media thickness |
| CVD | Cardiovascular disease |
| CVD/CVE | Cardiovascular diseases |
| CVE | Cardiovascular event |
| DES | Dagmar Else Slot; co-author of this paper |
| DM | Diabetes mellites |
| EJSW | Eveline Willems; co-author of this paper |
| FMD | Flow-mediated dilatation |
| GAW | Godefridus August van der Weijden; co-author of this paper |
| GRADE | Grading of recommendations assessment, development, and evaluation |
| HT | Hypertension |
| IHD | Ischemic heart disease |
| JBI | Joanna Briggs Institute |
| LEAD | Peripheral artery disease |
| LPMW | Lotte Phinè Marie Weijdijk; co-author of this paper |
| MGPS | Max Schoenmakers; first author of this paper |
| MI | Myocardial infarction |
| MOOSE | Meta-analysis of Observational Studies |
| NHMRC | National Health and Medical Research Council |
| NOS | Newcastle-Ottawa Scale |
| OR | Odds ratio |
| PAD | Peripheral artery disease |
| PerioD | Periodontitis, Periodontal disease |
| PROSPERO | Prospective register of systematic reviews |
| PVD | Peripheral vascular disease |
| RR | Relative risk |
| SR | Systemic review |
| TIA | Transient ischemic attack |
| TL | Tooth loss |
| UPS | Université Paul Sabatier |
| WHO | World Health Organization |
| ? | Unknown |

**Online Appendix S7**

JBI^55^ checklist.


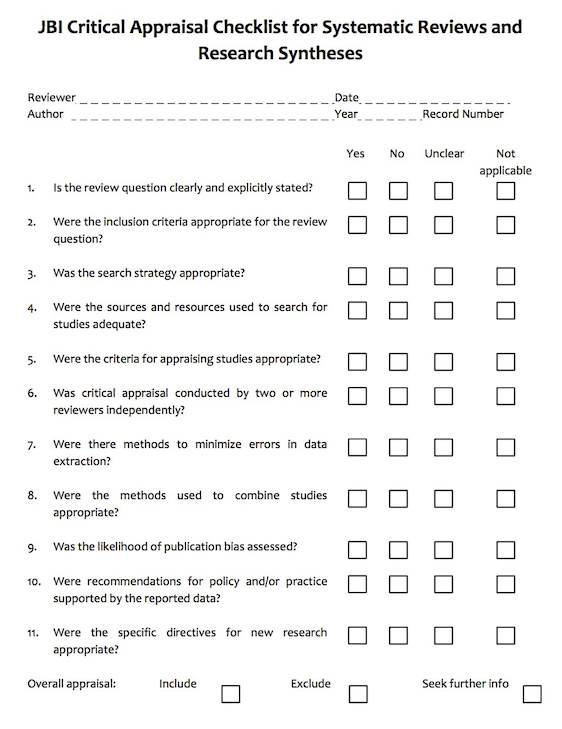


03/01/2023

2023

MGPS and LPMW

MGPS and LPMW

**Online Appendix S8**

MOOSE^56^ checklist.

| **Reporting Criteria** | **Reported (Yes/No)** | **Reported on Page No.** |
| --- | --- | --- |
| **Reporting of Background** |  |  |
| Problem definition | Yes | 5 |
| Hypothesis statement | No |  |
| Description of Study Outcome(s) | Yes | 6 |
| Type of exposure or intervention used | Yes | 6 |
| Type of study design used | Yes | 6 |
| Study population | Yes | 6 |
| **Reporting of Search Strategy** |  |  |
| Qualifications of searchers (eg, librarians and investigators) | Yes | 6 |
| Search strategy, including time period included in the synthesis and keywords | Yes | 6, Table 1 |
| Effort to include all available studies, including contact with authors | Yes | 7 |
| Databases and registries searched | Yes | 6, Figure 1 |
| Search software used, name and version, including special features used (eg, explosion) | Yes | 6 |
| Use of hand searching (eg, reference lists of obtained articles) | Yes | 6 |
| List of citations located and those excluded, including justification | Yes | Figure 1, Online Appendix S1 |
| Method for addressing articles published in languages other than English | No |  |
| Method of handling abstracts and unpublished studies | Yes | 7 |
| Description of any contact with authors | No |  |
| **Reporting of Methods** |  |  |
| Description of relevance or appropriateness of studies assembled for assessing the hypothesis to be tested | Yes | 7,8 |
| Rationale for the selection and coding of data (eg, sound clinical principles or convenience) | Yes | 8 |
| Documentation of how data were classified and coded (eg, multiple raters, blinding, and interrater reliability) | Yes | 8,9 |
| Assessment of confounding (eg, comparability of cases and controls in studies where appropriate | Yes | 9 |
| Assessment of study quality, including blinding of quality assessors; stratification or regression on possible predictors of study results | Yes | 9 |
| Assessment of heterogeneity | Yes | 8,9 |
| Description of statistical methods (eg, complete description of fixed or random effects models, justification of whether the chosen models account for predictors of study results, dose-response models, or cumulative meta-analysis) in sufficient detail to be replicated | Yes | 8,9 |
| Provision of appropriate tables and graphics | Yes | Table 1-6 |
| **Reporting of Results** |  |  |
| Table giving descriptive information for each study included | Yes | Table 2 |
| Results of sensitivity testing (eg, subgroup analysis) | No |  |
| Indication of statistical uncertainty of findings | Yes | 11 |
| **Reporting of Discussion** |  |  |
| Quantitative assessment of bias (eg, publication bias) | Yes | 11 |
| Justification for exclusion (eg, exclusion of non–English-language citations) | Yes | 11, Online Appendix S1 |
| Assessment of quality of included studies | Yes | 11 |
| **Reporting of Conclusions** |  |  |
| Consideration of alternative explanations for observed results | Yes | 15-20 |
| Generalization of the conclusions (ie, appropriate for the data presented and within the domain of the literature review) | Yes | 22 |
| Guidelines for future research | Yes | 21 |
| Disclosure of funding source | Yes | 1 |

**References**

1. Matthews D. Possible link between periodontal disease and coronary heart disease. *Evid Based Dent*. 2008;(9):8. doi:10.1038/sj.ebd.6400560

2. Froum SJ, Hengjeerajaras P, Liu KY, Maketone P, Patel V, Shi Y. The Link Between Periodontitis/Peri-implantitis and Cardiovascular Disease: A Systematic Literature Review. *Int J Periodontics Restorative Dent*. 2020;40(6):e229-e233. doi:10.11607/prd.4591

3. Cronin A. Periodontal disease is a risk marker for coronary heart disease? *Evidence-Based Dentistry* . 2009;(10):22. doi:10.1038/sj.ebd.6400634

4. Leng WD, Zeng XT, Kwong JSW, Hua XP. Periodontal disease and risk of coronary heart disease: An updated meta-analysis of prospective cohort studies. *Int J Cardiol*. 2015;201:469-472. doi:10.1016/j.ijcard.2015.07.087

5. Meurman JH, Sanz M, Janket SJ. Oral health, atherosclerosis, and cardiovascular disease. *Crit Rev Oral Biol Med*. 2004;15(6):403-413. doi:10.1177/154411130401500606

6. Choi H, Dey AK, Priyamvara A, et al. Role of Periodontal Infection, Inflammation and Immunity in Atherosclerosis. *Curr Probl Cardiol*. 2021;46(3):100638. doi:10.1016/j.cpcardiol.2020.100638

7. Baniulyte G, Piela K, Culshaw S. How strong is the link between periodontitis and stroke? *Evidence-Based Dentistry* . 2021;(22):10-11. doi:10.1038/s41432-021-0161-7

8. Dewan M, Pandit A, Goyal L. Association of periodontitis and gingivitis with stroke: A systematic review and meta-analysis. *Dent Med Probl*. 2023;61(3):1-9. doi:10.17219/dmp/158793

9. Leng Y, Hu Q, Ling Q, et al. Periodontal disease is associated with the risk of cardiovascular disease independent of sex: A meta-analysis. *Front Cardiovasc Med*. 2023;10:1-14. doi:10.3389/fcvm.2023.1114927

10. Guo X, Li X, Liao C, Feng X, He T. Periodontal disease and subsequent risk of cardiovascular outcome and all-cause mortality: A meta-analysis of prospective studies. *PLoS One*. 2023;18(9):e0290545. doi:10.1371/JOURNAL.PONE.0290545

11. Alwithanani N. Periodontal Diseases and Heart Diseases: A Systemic Review. *J Pharm Bioallied Sci*. 2023;15(Suppl 1):S72. doi:10.4103/JPBS.JPBS_517_22

12. Bahekar AA, Singh S, Saha S, Molnar J, Arora R. The prevalence and incidence of coronary heart disease is significantly increased in periodontitis: a meta-analysis. *Am Heart J*. 2007;154(5):830-837. doi:10.1016/j.ahj.2007.06.037

13. Blaizot A, Vergnes JN, Nuwwareh S, Amar J, Sixou M. Periodontal diseases and cardiovascular events: meta-analysis of observational studies. *Int Dent J*. 2009;59(4):197-209. https://pubmed.ncbi.nlm.nih.gov/19774803/

14. Dietrich T, Sharma P, Walter C, Weston P, Beck J. The epidemiological evidence behind the association between periodontitis and incident atherosclerotic cardiovascular disease. *J Clin Periodontol*. 2013;40:S70-84. doi:10.1111/jcpe.12062

15. Fagundes NCF, APCPSC A, Vilhena KFB, Magno MB, Maia LC, Lima RR. Periodontitis As A Risk Factor For Stroke: A Systematic Review And Meta-Analysis. *Vasc Health Risk Manag*. 2019;15:519-532. doi:10.2147/VHRM.S204097

16. Gao S, Tian J, Li Y, et al. Periodontitis and Number of Teeth in the Risk of Coronary Heart Disease: An Updated Meta-Analysis. *Med Sci Monit*. 2021;27:e930112. doi:10.12659/MSM.930112

17. Humphrey LL, Fu R, Buckley DI, Freeman M, Helfand M. Periodontal disease and coronary heart disease incidence: a systematic review and meta-analysis. *J Gen Intern Med*. 2008;23(12):2079-2086. doi:10.1007/s11606-008-0787-6

18. Janket SJ, Baird AE, Chuang SK, Jones JA. Meta-analysis of periodontal disease and risk of coronary heart disease and stroke. *Oral Surg Oral Med Oral Pathol Oral Radiol Endod*. 2003;95(5):559-569. doi:10.1067/moe.2003.107

19. Khader YS, Albashaireh ZS, Alomari MA. Periodontal diseases and the risk of coronary heart and cerebrovascular diseases: a meta-analysis. *J Periodontol*. 2004;75(8):1046-1053. doi:10.1902/jop.2004.75.8.1046

20. Lafon A, Pereira B, Dufour T, et al. Periodontal disease and stroke: a meta-analysis of cohort studies. *Eur J Neurol*. 2014;21(9):1155-1161, e66-7. doi:10.1111/ene.12415

21. Larvin H, Kang J, Aggarwal VR, Pavitt S, Wu J. Risk of incident cardiovascular disease in people with periodontal disease: A systematic review and meta-analysis. *Clin Exp Dent Res*. 2021;7(1):109-122. doi:10.1002/cre2.336

22. Leira Y, Seoane J, Blanco M, et al. Association between periodontitis and ischemic stroke: a systematic review and meta-analysis. *Eur J Epidemiol*. 2017;32(1):43-53. doi:10.1007/s10654-016-0170-6

23. Meregildo-Rodriguez ED, Robles-Arce LG, Chunga-Chévez EV, Asmat-Rubio MG, Zavaleta-Alaya P, Vásquez-Tirado GA. Periodontal disease as a non-traditional risk factor for acute coronary syndrome: a systematic review and meta-analysis. *Infezioni in Medicina*. 2022;30(4):501-515. doi:10.53854/liim-3004-4

24. Qin X, Zhao Y, Guo Y. Periodontal disease and myocardial infarction risk: A meta-analysis of cohort studies. *Am J Emerg Med*. 2021;48:103-109. doi:10.1016/j.ajem.2021.03.071

25. Sfyroeras GS, Roussas N, Saleptsis VG, Argyriou C, Giannoukas AD. Association between periodontal disease and stroke. *J Vasc Surg*. 2012;55(4):1178-1184. doi:10.1016/j.jvs.2011.10.008

26. Voinescu I, Petre A, Burlibasa M, Oancea L. Evidence of Connections Between Periodontitis and Ischemic Cardiac Disease - an Updated Systematic Review. *Maedica (Bucur)*. 2019;14(4):384-390. doi:10.26574/maedica.2019.14.4.384

27. Wang J, Geng X, Sun J, et al. The risk of periodontitis for peripheral vascular disease: a systematic review. *Rev Cardiovasc Med*. 2019;20(2):81-89. doi:10.31083/j.rcm.2019.02.52

28. Xu S, Song M, Xiong Y, Liu X, He Y, Qin Z. The association between periodontal disease and the risk of myocardial infarction: a pooled analysis of observational studies. *BMC Cardiovasc Disord*. 2017;17(1):50. doi:10.1186/s12872-017-0480-y

29. Zeng XT, Leng WD, Lam YY, et al. Periodontal disease and carotid atherosclerosis: A meta-analysis of 17,330 participants. *Int J Cardiol*. 2016;203:1044-1051. doi:10.1016/j.ijcard.2015.11.092

30. Sälzer S, Slot DE, Van Der Weijden FA, Dörfer CE. Efficacy of inter-dental mechanical plaque control in managing gingivitis - A meta-review. *J Clin Periodontol*. 2015;42(S16):S92-S105. doi:10.1111/jcpe.12363

31. Hidding JT, Beurskens CHG, Van Der Wees PJ, Van Laarhoven HWM, Nijhuis-van Der Sanden MWG. Treatment related impairments in arm and shoulder in patients with breast cancer: A systematic review. *PLoS One*. 2014;9(5). doi:10.1371/journal.pone.0096748

32. Scottish Intercollegiate Guidelines Network (SIGN). SIGN 50: A guideline developers’ handbook Methodology Checklist. Published online 1995. Accessed December 23, 2023. www.sign.ac.uk/guidelines/published/numlist.html.

33. Cho MK, Bero LA. Instruments for Assessing the Quality of Drug Studies Published in the Medical Literature. *JAMA*. 1994;272(2):101-104. doi:10.1001/JAMA.1994.03520020027007

34. Nuwwareh S, Sixou M. University Paul Sabatier critical appraisal tool for etiological studies. Published 2007. Accessed December 23, 2023. http://www.cnos.fr/publi/

35. Sterne JA, Hernán MA, Reeves BC, et al. ROBINS-I: a tool for assessing risk of bias in non-randomised studies of interventions. *BMJ*. 2016;355(4919):1-7. doi:10.1136/bmj.i4919

36. Singh A, Museedi AS, Grossman SA. Acute Coronary Syndrome. *StatPearls*. Published online July 11, 2022. Accessed December 23, 2023. https://www.ncbi.nlm.nih.gov/books/NBK459157/

37. Stang A. Critical evaluation of the Newcastle-Ottawa scale for the assessment of the quality of nonrandomized studies in meta-analyses. *Eur J Epidemiol*. 2010;22:603-605. doi:10.1007/s10654-010-9491-z

38. GRADE home. Accessed December 23, 2023. https://www.gradeworkinggroup.org/

39. National Health and Medical Research Council (NHMRC). *How to Review the Evidence: Systematic Identification and Review of the Scientific Literature. Handbook Series on Preparing Clinical Practice Guidelines Endorsed Production by Biotex (Canberra)*.; 1999.

40. Yu YH, Chasman DI, Buring JE, Rose L, Ridker PM. Cardiovascular risks associated with incident and prevalent periodontal disease. *J Clin Periodontol*. 2015;42(1):21-28. doi:10.1111/JCPE.12335

41. Dorn JM, Genco RJ, Grossi SG, et al. Periodontal disease and recurrent cardiovascular events in survivors of myocardial infarction (MI): the Western New York Acute MI Study. *J Periodontol*. 2010;81(4):502-511. doi:10.1902/JOP.2009.090499

42. Carallo C, Irace C, Tripolino C, et al. Time course analysis of brachial artery flow mediated dilatation in subjects with gingival inflammation. *Int Angiol*. 2014;33(6):565-572.

43. Hill AB. The Environment and Disease: Association or Causation? In: *Proceedings of the Royal Society of Medicine*. ; 1965:295-300.

44. Olivier J, May WL, Bell ML. Relative effect sizes for measures of risk. *http://dx.doi.org/101080/0361092620151134575*. 2017;46(14):6774-6781. doi:10.1080/03610926.2015.1134575

45. Chen H, Cohen P, Chen S. How big is a big odds ratio? Interpreting the magnitudes of odds ratios in epidemiological studies. *Commun Stat Simul Comput*. 2010;39(4):860-864. doi:10.1080/03610911003650383

46. Haraszthy VI, Zambon JJ, Trevisan M, Zeid M, Genco RJ. Identification of Periodontal Pathogens in Atheromatous Plaques. *J Periodontol*. 2000;71(10):1554-1560. doi:10.1902/JOP.2000.71.10.1554

47. Imamura T, Banbula A, Pereira PJB, Travis J, Potempa J. Activation of Human Prothrombin by Arginine-specific Cysteine Proteinases (Gingipains R) from Porphyromonas gingivalis. *Journal of Biological Chemistry*. 2001;276(22):18984-18991. doi:10.1074/JBC.M006760200

48. Aul P, Idker MR, Ary M, et al. Inflammation, Aspirin, and the Risk of Cardiovascular Disease in Apparently Healthy Men. *https://doi.org/101056/NEJM199704033361401*. 1997;336(14):973-979. doi:10.1056/NEJM199704033361401

49. Ebersole JL, Machen RL, Steffen MJ, Willmann DE. Systemic acute-phase reactants, C-reactive protein and haptoglobin, in adult periodontitis. *Clin Exp Immunol*. 1997;107(2):347-352. doi:10.1111/J.1365-2249.1997.270-CE1162.X

50. Ebersole JL, Cappelli D, Mathys EC, et al. Periodontitis in Humans and Non-Human Primates: Oral-Systemic Linkage Inducing Acute Phase Proteins. *Ann Periodontol*. 2002;7(1):102-111. doi:10.1902/ANNALS.2002.7.1.102

51. Sanz M, Marco del Castillo A, Jepsen S, et al. Periodontitis and cardiovascular diseases: Consensus report. *J Clin Periodontol*. 2020;47(3):268-288. doi:10.1111/JCPE.13189

52. Costerton J, Keller D. Oral periopathogens and systemic effects. *Gen Dent*. 2007;55(3):210-215. Accessed December 23, 2023. https://europepmc.org/article/med/17511362

53. Darré L, Vergnes JN, Gourdy P, Sixou M. Efficacy of periodontal treatment on glycaemic control in diabetic patients: A meta-analysis of interventional studies. *Diabetes Metab*. 2008;34(5):497-506. doi:10.1016/J.DIABET.2008.03.006

54. Vergnes JN, Sixou M. Preterm low birth weight and maternal periodontal status: a meta-analysis. *Am J Obstet Gynecol*. 2007;196(2):135.e1-135.e7. doi:10.1016/J.AJOG.2006.09.028

55. Aromataris E, Fernandez R, Godfrey CM, Holly C, Khalil H, Tungpunkom P. Summarizing systematic reviews: Methodological development, conduct and reporting of an umbrella review approach. *Int J Evid Based Healthc*. 2015;13(3):132-140. doi:10.1097/XEB.0000000000000055

56. Stroup DF, Berlin JA, Morton SC, et al. Meta-analysis of Observational Studies in Epidemiology: A Proposal for Reporting. *JAMA*. 2000;283(15):2008-2012. doi:10.1001/JAMA.283.15.2008
